# Classical vs. Non-Classical Cyclometalated Pt(II) Complexes

**DOI:** 10.3390/molecules27217249

**Published:** 2022-10-25

**Authors:** Luca Maidich, Maria I. Pilo, Jonathan P. Rourke, Guy J. Clarkson, Patrizia Canu, Sergio Stoccoro, Antonio Zucca

**Affiliations:** 1 Department of Chemical, Physical, Mathematical and Natural Sciences, University of Sassari, Via Vienna 2, 07100 Sassari, Italy; 2 School of Chemistry, Cardiff University, Main Building, Park Place, Cardiff CF10 3AT, UK; 3 Department of Chemistry, University of Warwick, Gibbet Hill Road, Coventry CV4 7AL, UK

**Keywords:** cyclometalation, rollover compounds, nitrogen ligands

## Abstract

Rollover cyclometalated complexes constitute a family of derivatives which differ from classical cyclometalated species in certain aspects. Various potential application fields have been developed for both classes of compounds, which have both similarities and differences. In order to uncover the relationships and distinctions between these two families of compounds, four Pt(II) cyclometalated complexes derived from 2-phenylpyridine (ppy) and 2,2′-bipyridine (bpy), assumed as prototypical ligands, were compared. For this study, an electron rich isostructural and isoelectronic pair of compounds, [Pt(N^C)Me(PPh_3_)], and an electron-poorer compound, [Pt(N^C)Cl(PPh_3_)] were chosen (N^C = ppy or bpy). DFT calculations, cyclic voltammetry, and UV-Vis spectra also helped to shed light into these species. Due to the presence of the more electronegative nitrogen in place of a C-H group, the rollover bpy-H ligand becomes a slightly weaker donor than the classical ppy-H ligand, and hence, generates (slightly) more stable cyclometalated complexes, lower energy frontier molecular orbitals, and electron-poorer platinum centers. On the whole, it was revealed that classical and rollover complexes have overall structural similarity, which contrasts to their somewhat different chemical behavior.

## 1. Introduction

The chemistry of cyclometalated complexes is a well-defined and important field of organometallic chemistry [1]. These species, due to their intrinsic stability related to the chelate effect, have found important applications in several fields, including catalysis [2], biomedicine [3], and innovative materials [4]. The large range of applications of these derivatives is related to the easy tunability of their properties. It is well known that, in cyclometalated complexes, the correlation between structure and properties can be remarkably high, and therefore, modification of the stereoelectronic parameters of the cyclometalated ligand, as well as those of co-ligands and metal, usually allows a refined modification of the overall properties of the complexes [5].

Among the many series of ligands studied, 2-phenylpyridine can be assumed to be a prototypical classical cyclometalating ligand that is able to give stable planar and stable five-membered cyclometalated complexes with a strong metal-C(sp^2^) bond [6]. Beyond classical cyclometalated complexes, variations on the theme have produced novel series of cyclometalated subclasses, such as that of the so-called “pincer complexes”, offering new, improved potential backbone modifications and consequent applications [7].

**Chart 1:**
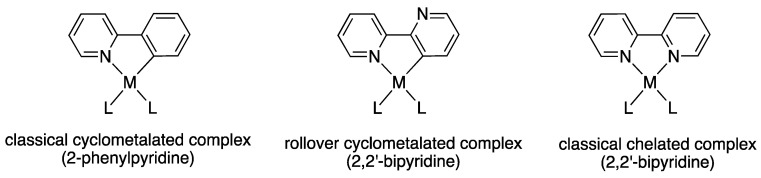
Classical and rollover complexes.

In the midst of the various families of conventional and unconventional cyclometalated complexes [8], the family of rollover cyclometalated complexes has attracted growing interest [9]. These compounds are derived from bidentate heteroaromatic ligands, such as 2,2′-bipyridine (Chart 1), which in some cases “decide” to rotate one of the heteroaromatic rings and undergo cyclometalation, activating a C-H bond “on the other side” (usually the one in the same relative position as the detached donor atom) [9,10].

**Scheme 1:**
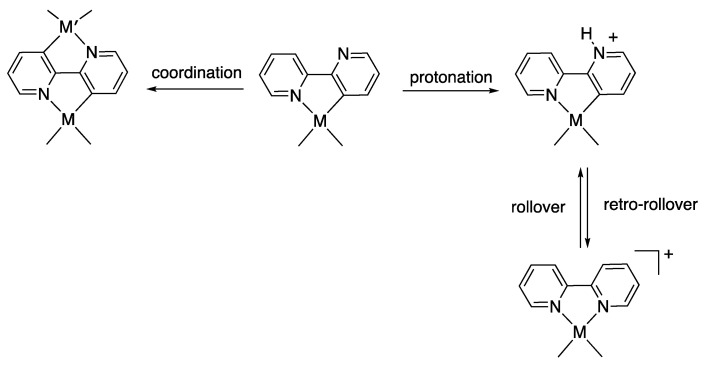
Reactions of rollover complexes not available for classical cyclometalated complexes.

The principal difference between classical and rollover complexes is the presence of the uncoordinated donor atom, usually a nitrogen. This presence extends the reactivity of the complex, leading to reactions not available for classical compounds, such as protonation [11] and coordination [12]. The acquisition of a hydrogen ion may allow a retro-rollover reaction [13], opening up new catalytic possibilities in the field of hydrogen transfer reactions (Scheme 1) [9,14]. In some cases the preference for the rollover form, over the classical form, may depend on something as simple as the solvent. [15] Due to the presence of the nitrogen atom, the rollover ligands belong to a family of “ligands with multiple personalities” [16].

Due to its relative novelty and peculiarity, in the last decade or so, rollover cyclometalation has achieved importance and found applications in organic synthesis [17,18,19] and catalysis [20,21,22,23,24,25]; rollover cyclometalated complexes have also shown potential applications as chemosensors [26], luminescent devices [27], and antitumor agents [28,29,30,31,32,33].

Following our longstanding interest in cyclometalation, in this paper, we aim to reveal the results from our investigations on the differences between classical and rollover cyclometalated complexes, by analyzing four Pt(II) complexes with cyclometalated 2-phenylpyridine and 2,2′-bipyridine of the general formula [Pt(N^C)X(PPh_3_)], with X = Me, Cl, in order to have both electron rich and electron poor complexes (Chart 1).

The two systems have a very similar five-membered, planar N^C metallacycle, with the only difference being the N/CH differentiation.

## 2. Results and Discussion

**Chart 2:**
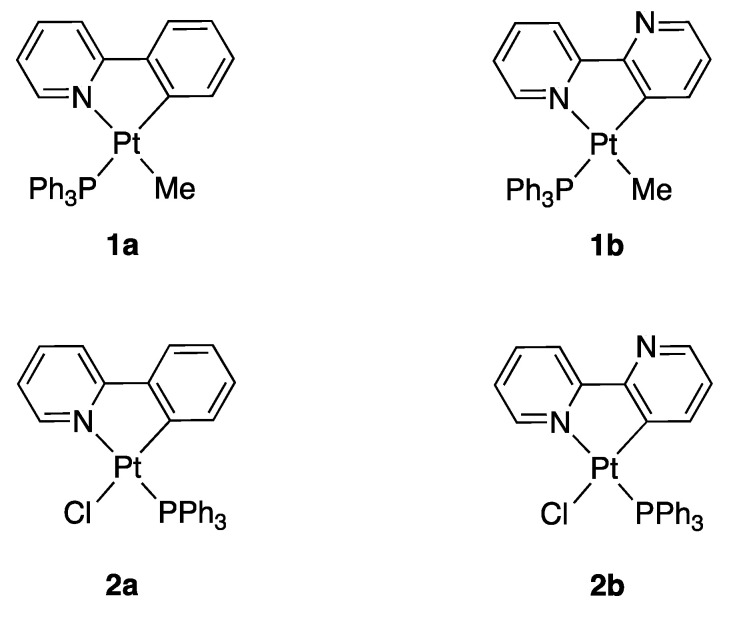
Cyclometaled complexes investigated in this study.

With the aim of comparing classical 2-phenylpyridine (ppy) complexes with the corresponding rollover-bipyridine (bpy) complexes, in this study, we report an investigation on four cycloplatinated complexes: [Pt(ppy-H)(PPh_3_)Me] (**1a**), [Pt(ppy-H)(PPh_3_)Cl] (**2a**), [Pt(bpy-H)(PPh_3_)Me] (**1b**), and [Pt(bpy-H)(PPh_3_)Cl] (**2b**) (Chart 2). It should be noted that the methyl complexes **1a** and **1b** are isostructural and isoelectronic (actually, they are also isoprotonic), as are the **2a**/**2b** couple. Compounds **1a/2a** and **1b/2b** differ by having a C-H in place of a N atom, whereas the electron poorer compounds **2a/2b** differ from the electron richer **1a/1b** by having an electronegative Cl in place of a methyl ligand. Compound families **1** and **2** also differ in that the stable, isolated stereoisomer has *trans*-P-Pt-C for **1a/1b** and *trans*-P-Pt-N for **2a/2b**, a consequence of the different *trans*-influence of the methyl and chloride ligands.

**Scheme 2:**
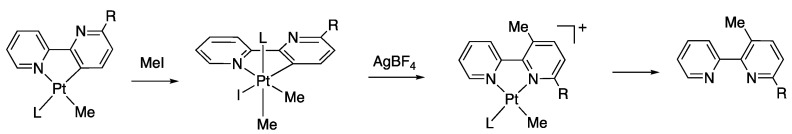
Reaction scheme for C^3^ functionalization of 2,2′-bipyridine by means of a rollover-retrorollover sequence.

Complexes **1a-b** and **2a-b** have been previously studied showing interesting properties. The reactivity of complexes **1a** and **1b** has common aspects but many important differences. Kinetic studies on the oxidative addition of MeI to **1a** and **1b** have shown a similar reaction mechanism, namely an S_N_2 reaction pathway, with large negative ΔS^‡^ values, affording the corresponding Pt(IV) complexes [Pt(ppy-H)(PPh_3_)(Me)_2_I], **3a**, and [Pt(bpy-H)(PPh_3_)(Me)_2_I], **3b** [34]. The authors found that **1a** in chloroform at 25 °C reacted nearly six times faster than **1b** with MeI (Scheme 2, first reaction); this behavior was ascribed to the presence of the electronegative nitrogen atom in **1b** reducing the electron density of platinum. As a consequence, the rollover bpy-H ligand can be assumed to be a slightly weaker donor than the classical ppy-H ligand, and the platinum center in **1a** electron-richer than in **1b**. The trend was confirmed with other phosphane ligands in place of PPh_3_.

Due to the presence of uncoordinated nitrogen, the rollover complex **1b** is able to follow reaction paths not available to **1a**, such as N-coordination (synthesis of dinuclear homo- and hetero-dimetallic species) [12], protonation [11], and retro-rollover reactions [13] as shown in Scheme 1. Furthermore, taking advantage of the uncoordinated nitrogen, complex **1b** has found application in organic synthesis, through an oxidative-addition/reductive-elimination reaction sequence which seems to be not available for **1a**, **2a**, and **2b** [17]. The reaction, passing through an intermediate cationic species ([Pt(N^N)(PPh_3_)Me]^+^, (Scheme 2), allows a rare C^3^ functionalization of 2,2′-bipyridine through a C(sp^3^)-C(sp^2^) coupling followed by a retro-rollover process, finally leading to 3-methyl-2,2′-bipyridine (Scheme 2).

From these data, it is evident that a richer chemical behavior is displayed by rollover species, whose free nitrogen adds new potentialities to those typically possessed by classical cyclometalated derivatives.

As for other applications, antitumor activity has been found and reported for **2a** [35,36] and **1b** [32], but not for **1a** and **2b**. Two complexes analogous to **1a** and **1b**, having a PPh_2_Allyl ligand in place of PPh_3_, i.e., [Pt(ppy-H)(PPh_2_Allyl)Me] and [Pt(bpy-H)(PPh_2_Allyl)Me], exhibit strong luminescence both in solution and in the solid state [37], with the phenylpyridine complex being brighter than the bipyridine one. In comparison, **1a** was found to be less bright.

### 2.1. Methyl Complexes **1a** and **1b**

Complex **1a** was first synthesized by Jamali, Nabavizadeh, and Rashidi in 2008 [38], and its solid-state structure was solved by Shahsavari and coworkers a few years later [37]. The X-ray crystal structure of **1a** was also previously solved by some of us [39] but is discussed here for the first time. The synthesis of the rollover complex **1b** was reported by some of us in 2009 [40], with its X-ray characterization in the solid state by crystallography being reported in 2014 [17].

**Figure 1:**
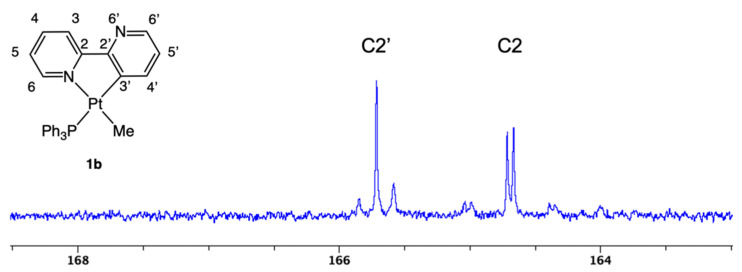
Expansion of the ^13^C NMR spectrum of **1b** showing C2 and C2′ signals.

In an attempt to analyze fundamental structural and thermodynamic differences between the two classes of compounds, first, we analyzed some spectroscopic and structural data for **1a** and **1b**. In order to better compare these species, we performed a detailed ^1^H and ^13^C NMR study on **1a**, by means of one- and two-dimensional NMR experiments (e.g., H,H DQF-COSY; NOESY; TOCSY; H,C HSQC; and HMBC, see experimental methods). First of all, the combined experimental data allowed us to fully assign ^1^H and ^13^C signals and correct some previous assignments, as well as resolve dubious points. As an example, quaternary C2 and C2′ signals in **1a** and **1b** have now unambiguously assigned by means of a long-range HMBC NMR experiment on **1a**. The signals are not very intense, deriving from quaternary carbons coupled to platinum and phosphorus. In the case of **1b**, the two peaks are close in the spectrum, appearing at 164.7 (^2^J_Pt-C_ = 48.5 Hz, ^3^J_P-C_ = 6.1 Hz) and 165.7 ppm (^2^J_Pt-C_ = 19.6 Hz). At first sight, it may be thought that the signal at 164.7 ppm, having the larger Pt-C coupling constant, should be assigned to C2′ (see Figure 1 below), due to the greater *trans*-influence of CH_3_ as compared with that of PPh_3_ (*trans* P-Pt-C3′ vs. *trans* N-Pt-CH_3_). However, in the same region, the ^13^C spectrum of **1a** shows only a signal at 167.1 (J_Pt-C_ = 69 Hz, ^3^J_P-C_ = 6.1 Hz) due to the pyridine ring, whereas the phenyl C1′ signal is shifted to 147.8 ppm (J_Pt-C_ = 10.5 Hz). NMR HSQC and HMBC heteronuclear spectra of **1a** unambiguously resolved the problem, the latter showing clear cross-peaks between C2 and H4, as well as between C2′ and H6′ and H4′.

As a consequence, NMR data allow us to conclude that the quaternary C adjacent to the coordinated nitrogen has the highest Pt-C and also P-C coupling constant values (no P-C coupling is seen for the quaternary carbon adjacent to the metalated aromatic carbon in spite of the trans P-Pt-C(sp^2^) coordination). This anomaly may be due to the sum of ^n^J + ^n+1^J coupling constants of opposite sign (see Figure 1 and Supplementary Materials Figure S1).

From the NMR spectroscopy, the two most important data to be considered are the ^195^Pt-^31^P and ^195^Pt-^13^C(sp^2^) coupling constants, which allow a rough correlation of the Pt-P and Pt-C bond strength.

In coordination chemistry, direct coupling constant values have often been used as an approximate measure of metal-ligand bond strengths, even though, theoretically, direct coupling constant values are mainly dependent on the Fermi-contact interaction between the nuclear moments and electron spins in s orbitals. Nevertheless, the evaluation of the prevailing parameter in the Fermi-contact interaction is not simple because many factors may contribute in similar or opposite amounts [41].

From a practical point of view, it has been generally found that direct coupling constants are roughly related to bond strengths, therefore, for example, ^1^J_Pt-P_ coupling constant values may be correlated to Pt–P bond strengths, with stronger and shorter Pt-P bonds having higher ^1^J_Pt-P_ values. Previous studies have shown that ^1^J_Pt-P_ coupling constant values may be more useful than Pt–P bond lengths obtained from X-ray analyses, which display, in some cases, very small bond length variations [42]. However, it should also be considered that coupling constant values depend on several factors, primarily the Fermi-contact term, and therefore, caution is needed in analyzing coupling constant data.

A comparison of NMR data shows that the ^195^Pt-^31^P coupling constant is higher in **1b** than in **1a** (**1a**, ^1^J_Pt-P_ = 2105 Hz; **1b**, ^1^J_Pt-P_ = 2229 Hz), potentially indicating a stronger Pt-P bond in **1b**, and hence, a reduced donor ability of the C(sp^2^) donor in the rollover ligand; however, the ^195^Pt-^13^C direct coupling constant in **1b** is also slightly larger (**1a**, ^1^J_Pt-C_ = 956 Hz; **1b**, ^1^J_Pt-C_ = 970 Hz), complicating the picture. In addition, *trans* ^2^J_P-C_ coupling constants in **1a** and **1b** are almost equivalent (ca 120 Hz). In contrast, ^31^P NMR chemical shift values, as well as ^1^H NMR data, do not seem to be significantly different.

The same information (J_Pt-C_, J_P-C_) can be obtained, with a lower degree of resolution, from two-dimensional HMBC spectrum, which, therefore, can be used when the elusive satellites of the quaternary Pt-coordinated carbon signal cannot be obtained through a direct ^13^C NMR spectrum.

**Chart 3:**
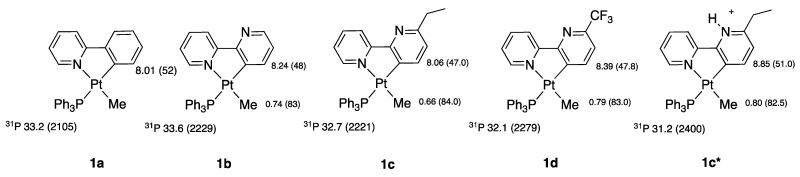
Selected ^1^H and ^31^P NMR data for some cyclometalated and rollover species. Chemical shifts are given in ppm, and coupling constants, in parentheses, are given in Hertz.

In order to better understand these data, we analyzed ^1^J(^195^Pt-^31^P) and ^1^J(^195^Pt-^13^C) values in some related rollover complexes. First, we analyzed [Pt(bpy^6Et^-H)(PPh_3_)Me], **1c**, and [Pt(bpy^6CF3^-H)(PPh_3_)Me], **1d** (see Chart 3, bpy^6Et^ = 6-ethyl-2,2′-bipyridine and 6 bpy^6CF3^ = trifluoromethyl-2,2′-bipyridine) in order to compare other electron rich and poor rollover donors. The ^1^J_Pt-P_ value in **1c**, i.e., 2221 Hz, is slightly smaller than that in **1b**, showing that the electron-releasing effect of an ethyl group in *para* position to the metalated carbon follows the observed trend, even though being very small; the higher value (2279 Hz) found for **1d** is in line with the electron withdrawing effect of a CF_3_ substituent. In agreement, nitrogen protonation in **1c** produces a mesoionic species 1c* whose elevated ^1^J_Pt-P_ value (2400 Hz) accounts for a formally neutral bipyridine, which is the worst donor.

As for ^1^J(^195^Pt-^13^C) values, data are not always available, due to the challenging problem of finding the elusive ^195^Pt satellites of quaternary carbon atoms, in sometimes not so soluble compounds. We compared ^1^J(^195^Pt-^13^C) data for some related corresponding DMSO complexes: **4d** [43] ([Pt(bpy6^CF3^-H)(DMSO)Me], δ 149.5 ppm, ^1^J_Pt-C_ = 1100 Hz) with 1090 Hz (δ 145.1 ppm), **4b** ([Pt(bpy-H)(DMSO)Me]), and 1071 Hz (δ 152.1 ppm) and in **4a** ([Pt(ppy-H)(DMSO)Me]) [44] (in the experimental section, however, the authors reported different data, i.e., δ 150.4 ppm and ^1^J_Pt-C_ = 1062.8 Hz)). As for ^3^J_Pt-H_ couplings for the coordinated DMSO (to be considered very carefully, being three-bonds couplings), the values are: [Pt(ppy-H)(DMSO)Me], **4a**, ^3^J_Pt-H_ = 17.6 Hz; [Pt(bpy-H)(DMSO)Me], **4b**, ^3^J_Pt-H_ = 18.3 Hz; [Pt(bpy^6CF3^)(DMSO)Me], **4d**, ^3^J_Pt-H_ = 18.5 Hz.

Overall, the observed trend shows higher ^1^J(^195^Pt-^13^C) and ^1^J(^195^Pt-^31^P) values corresponding to the lower (sigma) donor ability of the C-donor of the cyclometalated ligand.

#### X-ray Analysis

**Figure 2:**
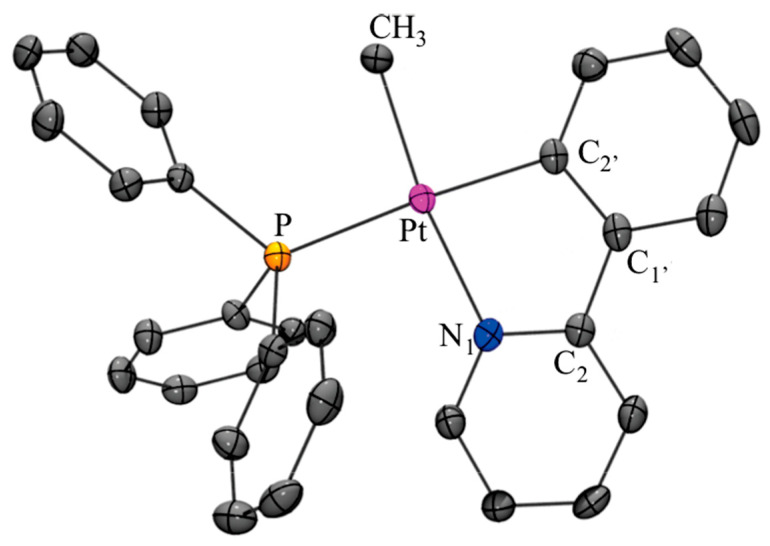
ORTEP view of complex **1a**. Ellipsoids are drawn at the 30% probability level.

Crystals of **1a** suitable for X-ray analysis were obtained by slow evaporation of a CH2Cl2 solution. An ORTEP drawing of the complex is shown in Figure 2, and selected bond lengths and distances are listed in Table S1. Crystallographic parameters concerning data collection and structure solution are provided in the experimental Materials and Methods Section and in the Supplementary Materials. Complex **1a** crystallizes in the monoclinic crystal system with a P21/c space group, the same found in the previously reported X-ray structure **1a′** [37].Four molecules are present in the unit cell and no unusual interactions are present in the crystal packing, apart from a short contact of 2.895 Å between Pt and the *meta* H of PPh_3_ of an adjacent molecule.

As expected, the platinum atom displays a square-planar coordination, with a slight tetrahedral distortion and no unusual bond distances and angles.

Bond distances and angles around the metal in the two crystal structures of **1a** (**1a**, reported here, and **1a′**, reported in [36] appear not to be identical, with a maximum difference in the Pt-C(sp^2^) bond distance (2.011(3) vs. 2.044(9) Å). The difference barely lies inside 3 e.s.d.s, but this divergence between different X-ray resolutions of the same complex suggests that the comparison of bond lengths in different compounds differing 0.02–0.03 Å may not be significant. It should be mentioned, however, that the structures of **1a** were recorded at different temperatures, i.e., 150 and 298 K for **1a** and **1a′**, respectively.

Indeed, when comparing classical **1a** and rollover **1b** structures, bond and angle values appear very similar, with the former **1a″** X-ray resolution being the most different of the three. On the whole, no significant differences in bond lengths around the metal can be outlined, and the influence of the metalated ligands ppy-H and bpy-H on the Pt-N, Pt-P, and Pt-C bond lengths appears to be negligible.

As for the angles, some differences are present in the structures. In particular, the N-Pt-P angle is slightly larger in **1b** (97.91(6)°) than in **1a**, (i.e., 96.69(8) and 96.82(19)° in **1a′** and **1a″**, respectively).

Even though bond distances and angles are very similar in **1a** and **1b**, the distortion parameters may play a role in differentiating the two complexes.

**Figure 3:**
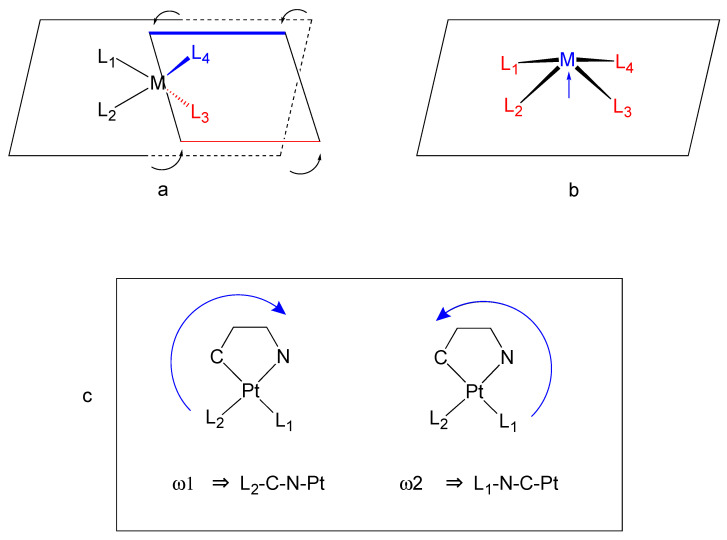
Distortions in square planar complexes: (**a**) Tetrahedral; (**b**) square pyramidal. (**c**) Sequence of atoms used for improper torsion angle measurements. Same sign of ω1 and ω2, tetrahedral distorsion; different signs of ω1 and ω2, square pyramidal distorsion.

The distortion in square planar complexes with chelated planar aromatic ligands such as 2,2′-bipyridine has been described by a number of authors; several different modes of distortion have been analyzed and reported, from the simple tetrahedral and square pyramidal distortions, related to the four Pt-L bonds (Figure 3), to internal distortions of the bidentate ligand (typically 2,2′-bipyridine or 1,10-phenanthroline). In the latter case, bowing, twisting, bending, and S-shaped distortions have been reported [45,46,47].

In 2003, Pérez and coworkers quantified the distortion in square planar complexes towards tetrahedral and square pyramidal coordination making use of improper torsion angles [48]. Their method is particularly useful for cyclometalated complexes. As an example, in square planar [M(N^C)L_1_L_2_] complexes (L_1_ *trans* to C; L_2_ *trans* to N), the L_1_-N-C-M and L_2_-C-N-M improper torsion angles (ω1 and ω2, respectively) were used to quantify the deviation from the planar coordination in a tetrahedral distortion sign ω1 = sign ω2, whereas in a square pyramidal distortion sign ω1 ≠ sign ω2. In an ideal square planar geometry both values should equal zero. Therefore, in complexes **1a** and **1b** we calculated improper torsion angles for P-N-Csp^2^-Pt and Csp^3^-Csp^2^-N-Pt (see Table 1). The values found indicated tetrahedral distortion for all **1a** and **1b** structures, with higher distortion values displayed by **1b**.

We came to the same conclusions when analyzing the position of the P and C(sp^3^) atoms towards the cyclometalated plane (Pt, N and C(sp^2^) atoms, Pl_1_, Figure 3); in the three structures, the P and C(sp^3^) atoms lie on different parts of space (one above and the other one below the cyclometalated plane), confirming tetrahedral distortion. However, the distortion found in **1b** is far greater that in **1a′/1a″** (**1b**: C 0.227 Å below, P 0.233 Å above; **1a′**: C 0.028Å above, P 0.180 Å below), in addition, the C(sp^3^) and P atoms are reversed in position in **1b** with respect to **1a′/1a″**.

**Figure 4:**
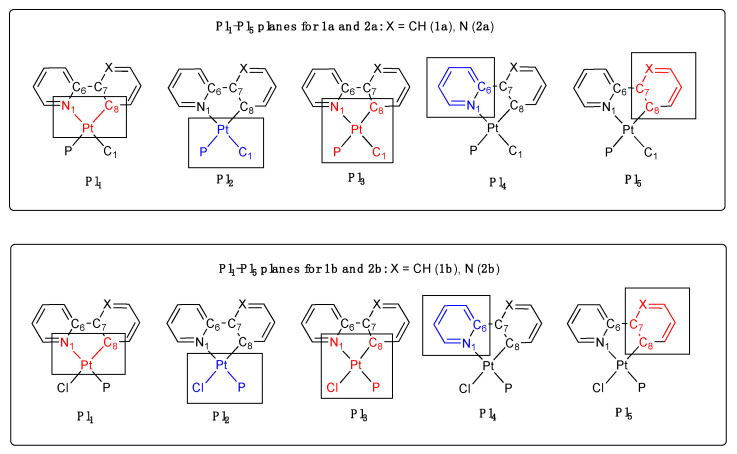
Planes used for the structural discussion of **1a-b** and **2a-b** with numeric schemes.

In order to compare distortions in **1a** and **1b**, we considered five different planes in the molecules: the above mentioned Pl_1_, the Pt-P-C_1_ plane (Pl_2_); the mean Pt-N_1_-C_8_-P-C_1_ square planar plane (Pl_3_); the mean N_1_-bonded pyridine ring Pl_4_; the mean C_8_-bonded pyridine; the phenyl ring (Pl_5_) (see Figure 4).

In an ideal square planar geometry, all these atoms should be coplanar and the dihedral angles between them should be zero. Selected data are reported in Table 1.

The dihedral angles between Pl_1_ and Pl_2_ confirm the higher distortion in **1b** with respect to **1a** and **1b**, with a dihedral angle of 8.48° (**1b**) vs. 4.55° (**1a′**) and 4.13° (**1a″**).

Considering the best plane for the cyclometalated ring (Pl_3_, Pt_1_–N_1_–C_6_–C_7_–C_8_ atoms), complex **1b** presents a C1 atom well above this plane of 0.354 Å, whereas in **1a** it is the P1 atom that has the furthest deviation from this plane (0.302 and 0.301 Å, **1a′** and **1a″**, respectively).

The cyclometalated 2-phenylpyridine and 2,2′-bipyridine ligands are not completely planar showing a dihedral angle between the two aromatic rings (Pl_4_ and Pl_5_) of 8.79 (**1a**), 6.60 (**1a′**), and 6.63 (**1b**) degrees. These two aromatic rings also show significant deviations from the best square planar plane Pl_3_ (Pt-N_1_-C_8_-P_1_-C_1_), which reaches a maximum of 10.82° for the N-coordinated pyridine ring in **1b**.

On the whole, these data show that **1a** and **1b** are very similar with regard to bond distances and angles, but have, in contrast, different distortion parameters. In particular, a higher tetrahedral distortion is displayed by the rollover complex **1b**.

### 2.2. Chlorido Complexes **2a** and **2b**

The electron poorer chloride complexes **2a** and **2b** have been isolated only in the *trans* P-Pt-N configuration, in agreement with *trans* influence rules. To the best of our knowledge, not many examples of reactivity for these species have been reported in the literature, apart from simple ligand substitution reactions.

Complex **2a** can be obtained both by an open-bridge reaction of the corresponding chlorido dimer [Pt(N^C)Cl]_2_ with PPh_3_ [35] or a substitution reaction from the DMSO complex [Pt(N^C)(DMSO)Cl] [36]. Complex **2a** has shown interesting biological activity, demonstrating cytotoxic activity in vitro against the mouse lymphoid leukæmia cell line L1210 [35]. and against three human cancer cell lines [36]. Emissive properties of **2a** and some related compounds have also been reported [49,50].

In contrast, **2b** was first reported by some of us in 2009 [40] and no biological activity has hitherto been described. Complex **2b** constitutes the starting point for the synthesis of homo- and hetero-dinuclear di-cyclometalated complexes [12,40] taking advantage of the second, uncoordinated nitrogen atom, an opportunity not available for classical complexes such as **2a**.

Analysis of ^31^P NMR data for **2a** and **2b** shows that the ^195^Pt-^31^P coupling constant is larger in **2a** than in **2b** (^1^J_P,Pt_ = 4321.5 and 4285 Hz, respectively) reflecting an inverse trend with respect to **1a** and **1b**. It is generally agreed that *cis*-influence is smaller than *trans*-influence with corresponding ligands, and its evaluation may not be simple. The estimation of *cis*-influence may be obtained from direct ^195^Pt-^31^P coupling constants, whereas bond lengths obtained from X-ray analyses may fail to detect the small *cis*-influence because of very small Pt–P bond lengths variations [42].

The crystal structure of **2a** has been solved and reported several times. In 2016, Shahsavari and coworkers reported, for the first time, the X-ray structure of **2a** (CCDC database identifier 1457812) [51]. One year later, Tunik and coworkers reported the X-ray structure of the complex, finding polymorphism in the solid state with two types of crystals with different unit cell parameters (CCDC database identifiers 1505457 and 1505459) [49]. The X-ray crystal structure of **2a** was also deposited by some of us in 2017 [39](CCDC database identifier 1546694) and is discussed here for the first time. In contrast, the crystal structure of **2b** has been reported only once [40].

The crystal structure of complex **2a** reported here (hereinafter 2a′) will be compared with the following previously reported structures: **2a″** (reported by Shahsavari et al., CCDC database identifier 1457812), **2a‴**, and **2a′′′′**, (two reported by Tunik et al. [49], CCDC database identifiers 1505457 and 1505459, respectively).

**Figure 5:**
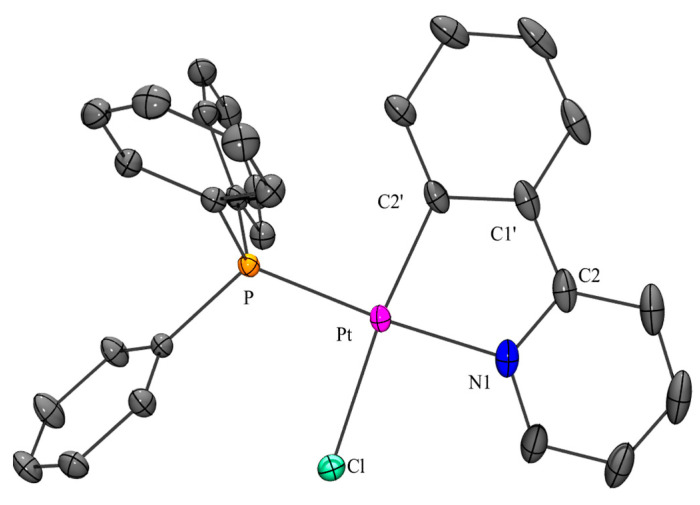
ORTEP view of complex **2a′**. Ellipsoids are drawn at the 50% probability level.

Complex **2a′** crystallizes in the triclinic P-1 space group; there are two complexes in the unit cell related by a crystallographic centre of inversion. An ORTEP view of complex **2a′** is reported in Figure 5. Geometrical parameters, i.e., bond distances and angles, are reorted in the Supplementary Materials (Table S2). The structure confirms the square planar geometry with square-pyramidal distortion, as showed by the sign of ω1 and ω2 improper torsion angles (see later in the discussion).

The structure of **2a′** reveals a π stacking between the cyclometalated phenylpyridine of two neighboring complexes, with closest atomic contact between the C2 on one phenylpyridine and the C5 in the symmetry-related one of 3.309(2) Å.

In addition, the structure evidences a short Cl-H6 contact, 2.592 Å, well below the sum of the van der Waals radii (3.00 Å, [52]). This interaction may account for the noteworthy downfield shift often observed in cyclometalated complexes for H6′ protons when there is a neighboring chloride ligand [53].

Examination of bond lengths and angles of the four structures of **2a** show that all the metrics are similar, differing by less than 3 e.s.d.s, with the only exception being the P-Pt-Cl angle, which is larger in **2a′′′′**, i.e., 92.31(5) vs. 91.55(2) in **2a′** or 91.67(8) in **2a″**.

Worthy of note, in the two structures of **2a** reported by Shahsavari and coworkers, the Pt-P bond lengths fall outside 3 e.s.d.s (2.2250(1) vs. 2.234(1) Å), even if this difference is only on the second decimal digit, as discussed earlier. It warns us that in the successive comparison between **2a** and **2b** we cannot consider such differences as significant.

The comparison of **2a** with **2b** shows that all the complexes crystallize in the triclinic crystal system. Complex **2b** presents two crystallographically independent molecules in the asymmetric unit (**2b′** and **2b″**).

The comparisons of bond lengths and angles between **2a** and **2b** do not show significant differences, with the exception of the N1-Pt-Cl angle larger in **2a** (**2a**, e.g., 92.60(4–8) and 92.5(1–2)°; **2b**, 90.77(6) and 91.51(6)°).

For the geometrical distortion from square planar coordination, we defined for **2a** and **2b** the same Pl_1_–Pl_5_ planes previously described (see Figure 4). In contrast with **1a** and **1b, 2a** structures show different types of distortions: **2a′**, **2a″**, and **2a′′′′** structures show opposite signs for ω1 and ω2 improper torsion angles (e.g., ω1 −1.34 and ω2 + 1.00 for **2a′**) indicative of square pyramidal distortion, whereas the same sign was found in **2a‴** and both **2b** molecules (e.g., −1.09 and ω2.35), indicative of a tetrahedral distortion. As for the position of Cl and P atoms with respect to the Pl_1_ plane, the observed data confirm the square pyramidal distortion for **2a′**, **2a″**, and **2a′′′′** (both above or below the plane) and the tetrahedral distortion for **2a‴** and **2b**. The distortion is, in some cases, very small (e.g., in **2a′′′′**, Cl and P atoms located very close to the plane: Cl-0.058 Å and P-0.072 Å, see Table 1), and more evident in other cases, such as in **2a‴**, with a visible tetrahedral distortion a larger P-Pt-Cl angle (92.31°, as previously mentioned).

As for distortion regarding the aromatic rings of the cyclometalated ligands, **2a′**, **2a″** and **2a‴** appear similar, with a pyridine ring almost coplanar to the coordination plane Pl_3_ (dihedral angles of only 0.7–0.8°) and an out-of-plane phenyl ring (ca 5.2–5.6°). In contrast, **2a′′′′** appears different, with both rings well distant from coplanarity (6.91 and 4.43° to the coordination plane).

This data show that the same compound may crystallize with different modes and noteworthy degrees of distortion.

#### 2.2.1. DFT Calculations

A series of DFT calculations were performed to further investigate the relationships between the methyl complexes **1a**/**1b** and the chloride complexes **2a**/**2b**.

Before discussing the details of the results, it is worth mentioning that the systems are subject to different degrees of freedom and, for the scope of the analysis included in this paper, we focused our attention on the main features of the complexes.

**Figure 6:**
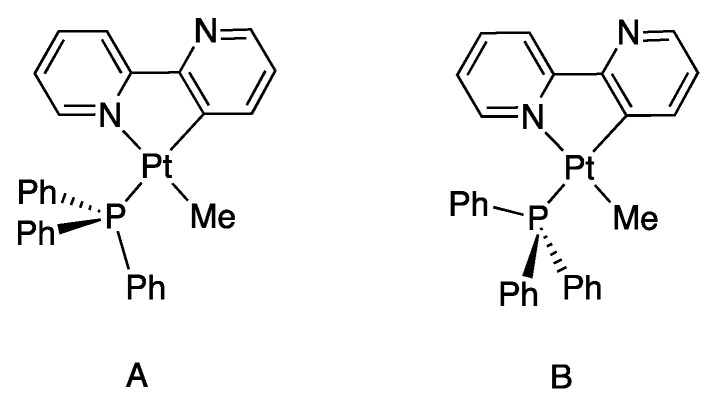
Complex **1b** rotamers **A** and **B** (PPh_3_ rotation).

We located the minimum energy geometries by performing potential energy surface (PES) scans rotating the PPh_3_ and, specifically for **1a**/**1b**, the methyl ligands. The rotation of PPh_3_ leads to a couple of rotamers for each investigated complex: one rotamer, with two phenyl rings on the same side of the N-coordinated pyridine ring, placed above and below the cyclometalated plane (conformer A, Figure 6) and the second rotamer rotated by 60°, with a P-C bond on the same plane of the cyclometalated ring (conformer B).

The analysis of the energy profiles obtained for **1** and **2** shows that the most stable conformation is the one indicated as A, i.e., the one always found in X-ray crystal structures of analogous complexes, where the PPh_3_ ligand prefers to make room for the adjacent pyridine ring rather than for the coordinated methyl group (or chloride ligand, in the case of **2a** and **2b**). Looking at the values collected in Table 2, it is clear that the chloride complexes **2a** and **2b** display a higher energy difference between the two rotamers as compared with **1a** and **1b**, with straightforward implications for the equilibrium mixture.

The higher stabilization values found for the chlorido complexes may be due to different Pt-P bond lengths in **1a-b** and **2a-b**, due to P-*trans*-C and P-*trans*-N coordination, respectively. The shorter Pt-P distance in **2a** and **2b**, 2.22–2.23 Å as compared with 2.29–2.30 Å in **1a** and **1b** should result in stronger P-Ph—cyclometalated ligand interactions and higher destabilization of the less stable conformer.

We also verified that, for the methyl complexes **1a** and **1b**, the isomer having a P-*trans*-C arrangement is the thermodynamically favored one, in agreement with the experimental findings, over the P-*trans*-N which leads to two carbon atoms in relative *trans* position. By contrast, for chloride complexes **2a** and **2b**, the P-*trans*-N was found to be the favored one, also in this case in agreement with experimental data. A comparison of the energies between similar conformers shows that chloride complexes display a higher difference as compared with the methyl ones, specifically 30–40 kJ/mol for the former and 13–20 kJ/mol for the latter.

Qualitative information on the energy profile with the obtained values for the two maxima found during the PES scan for the Pt-PPh_3_ and Pt-CH_3_ bond rotation are collected in Table 3.

Analysis of bond angles and distances at the equilibrium geometries and comparison with the reported crystal structures is reasonably good and in line with the experimental error (see discussion above). Mean absolute errors in which the heavy metal atom is present are, in all cases, below 0.03 angstroms for distances and less than 2 degrees for angles; differences in other geometrical parameters are even smaller. Using a more conservative approach as regards the evaluation of errors, i.e., using the square root of the averaged squared errors, we observe a slight increase in the values but the overall situation does not change and the errors are of the same order of magnitude as the experimental ones.

**Figure 7:**
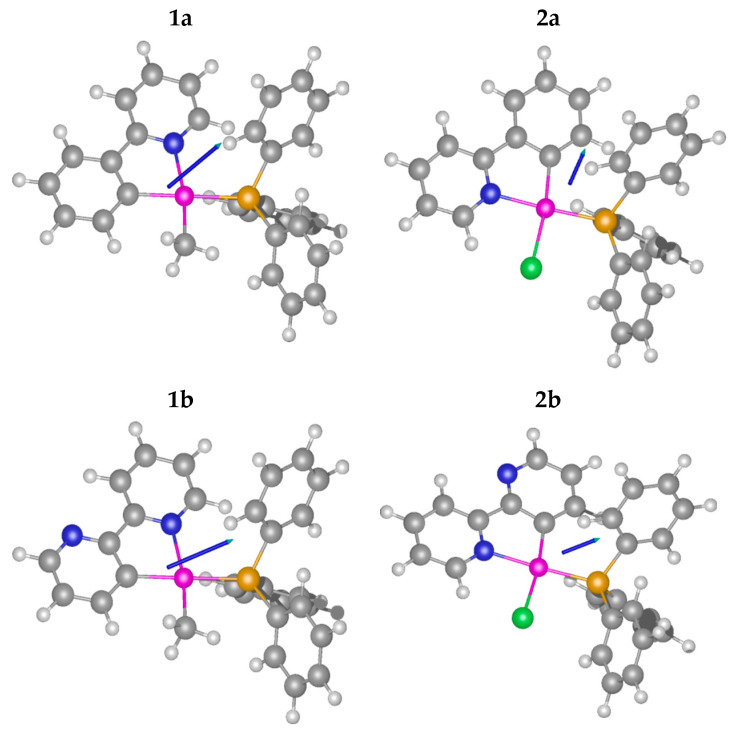
Calculated dipole moments for **1a-b** and **2a-b**.

Dipole moments of the equilibrium geometries were investigated both *in vacuo* and in CH_2_Cl_2_ solvent and they are similar for chlorides and methyl complexes. In gas phase, on the one hand, for **1a**/**1b**, the dipole is around 5.8 Debye and is directed towards the half of the molecule with the PPh_3_ and the N-bonded cyclometalated ligand; on the other hand for **2a**/**2b** the dipole moment is lower in absolute value, i.e., 2.97 for **2a** and 3.21 for **2b**, and points in the direction of the PPh_3_ and the C-bonded metalated ring (Figure 7).

**Figure 8:**
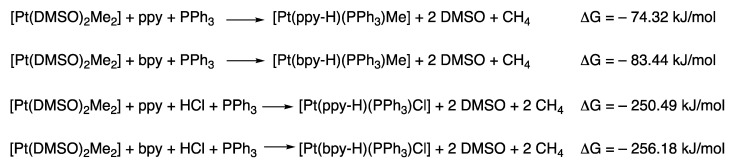
Overall reaction for the synthesis of **1a-b** and **2a-b** with ZPE-corrected Δ*G* values.

In addition, in order to compare the relative stability of **1a/1b** and **2a**/**2b**, we also evaluated the *ZPE*-corrected Δ*G* values for the overall cyclometalation reactions (Figure 8).

In this way, after subtraction of the same reagents and products, we can compare the relative stability of the cyclometalated complexes towards the corresponding free cyclometalating ligand.

A comparison of the calculated Δ*G* values for **1a** and **1b**, −74.32 and −83.44 kJ/mol, respectively, indicates that the rollover complex **1b** is more stable than the corresponding 2-phenylpyridine complex **1a**, relative to the starting ligand, by ca. 9 kJ/mol. Furthermore, also in the case of **2a** and **2b**, we find a similar situation with ZPE-corrected Δ*G* values of −250.49 and −256.18 kJ/mol for **2a** and **2b**, respectively. In all cases, we considered the more stable *trans*-conformation for free 2,2′-bipyridine.

Classical five-membered cyclometalated complexes have always been considered to be particularly stable due to the chelate effect; we can now assume that corresponding cyclometalated rollover complexes, showing this higher intrinsic stability, may be even more stable than the corresponding 2-phenylpyridine complex, at least for Pt(II).

Aiming at a deeper look into the electronic structure of the complexes, we performed an extended charge decomposition analysis (ECDA) to investigate the interaction of selected fragments of the four complexes. To simplify matters we chose to investigate two specific interactions: (a) the neutral PPh_3_ ligand and (b) the cyclometalated ligand, both with their respective remaining part of the complex.

In the case of PPh_3_, the donation from the neutral ligand to the other fragment of the complex is greater in the chloride complexes, accounting for a net 0.32–0.33 electrons in **1a**/**1b** and 0.46 electrons in **2a**/**2b**. This is in line with the different trans-influence of sp^2^ nitrogen and carbon donors. Donation ability is slightly greater in rollover complexes (0.3330 electrons in **1b** vs. 0.3237 electrons in **1a** and 0.4652 electrons in **2b** vs. 0.4565 electrons in **2a**), in agreement with a lower donating ability of the bpy-H ligand. All these data are in agreement with ^1^J(Pt-P) values found in the NMR spectra.

As regards the cyclometalated ligand, the donation to the other part of the complex, i.e., the formally cationic [Pt(Me)(PPh_3_)] fragment, is higher, accounting for 0.93 electrons in **1a**/**1b** and 1.03 in **2a**/**2b**. The data confirm that the phenylpyridine cyclometalated ligand is a slightly better donor than the rollover one, donating 0.9335 (**1a**) vs. 0.9304 (**1b**) electrons in the methyl complexes and 1.0335 (**2a**) vs. 1.0288 (**2b**) electrons in the chloride derivatives. Complete data are available in the Supplementary Materials (Tables S8 and S9).

An additional step with the LMO-EDA analysis was performed to investigate the single components, accounting for the total interaction energy for the complexes. Interaction energies are reported in Table 4, while full data can be found in the Supplementary Materials (Table S10).

The analysis of the results shows an overall good agreement with *trans*-influence principles for PPh_3_ and anionic ligands. In particular, it is worth noting that (**a**) the interaction energy of the anionic ligand (Me or Cl) with bpy complexes is always higher than analogous ppy complexes and (**b**) interaction energy of PPh_3_ is higher for chloride complexes **2a**/**2b** than for methyl complexes **1a**/**1b**, well in line with NMR data. In addition, the interaction energy of the cyclometalated ligand is higher for ppy than for bpy in line with its higher donor ability.

**Figure 9:**
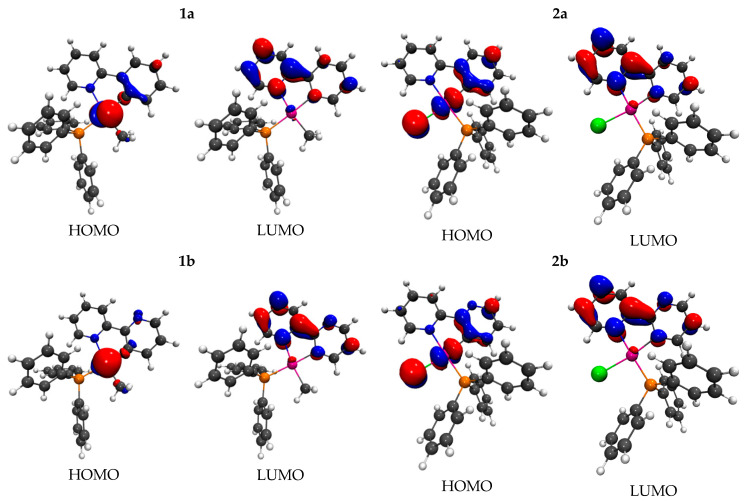
Frontier molecular orbitals for complexes **1a-b** and **2a-b**.

An analysis of the molecular orbitals was performed in the interval from HOMO-10 to LUMO+10; in Figure 9, we report HOMO and LUMO for the four complexes. All the complexes share a very similar LUMO which is mainly located on the cyclometalated ligand with a partition of ca. from 65% to 25% between N-bonded and C-bonded rings.

Looking at the occupied Mos, we can group the four compounds in two couples based on the anion. On the one hand, chloride complexes have the HOMO almost equally divided between the metal center, the cyclometalated ligand, and the chloride; on the other hand, methyl complexes have HOMOs mainly located on metal center and metalated ligand (Supplementary Materials Table S7).

A comparison of similar molecular orbitals between bpy and ppy cyclometalated complexes shows that those of the former, i.e., **1b**/**2b**, are generally ca. 10 kJ/mol lower in energy with the interval spanning from 5 to 15 kJ/mol (related to the higher electronegativity of nitrogen vs. carbon). The MO identifiable with the lone pair on the uncoordinated nitrogen is HOMO-3 in **1b** and HOMO-4 in **2b** with the latter being ca. 47 kJ/mol more stable. Last, but not least, it is interesting to note that the energy of the HOMO in methyl complexes **1a**/**1b** is lower as compared with the corresponding chlorides by ca. 9 and 7 kJ/mol, respectively.

The TD-DFT calculations were performed on the minimum energy isomer/rotamer for each compound looking for the 20 lowest excitations and a reasonably good agreement between theory and experiment was found. HOMO-LUMO transitions, evaluated applying SMD modeling with CH_2_Cl_2_ parameters to the optimized geometry *in vacuo*, are correctly reproduced, as summarized in Table 5; assignment of the peaks is also supported by the calculations (see Tables S11–S14 in Supplementary Materials for details).

#### 2.2.2. Electronic Spectroscopy and Electrochemical Behavior

Further information on relationships and differences between the two classes of complexes can be deduced from electrochemical and UV investigations.

##### **1a** and **1b**

Cyclic voltammetry at a Pt electrode of **1a** and **1b** shows a reproducible, irreversible anodic process at Ep,a = 0.83 and 0.95 V, respectively, at a potential scan rate equal to 100 mV s^−1^. Peak potential shifts at more anodic values and peak current increases at increasing the potential scan rate between 20 and 500 mV s^−1^. Potential values suggest the oxidation of the bpy derivative is more difficult than that of the ppy derivative, thus, confirming the higher stability of **1b** indicated by the ΔG values previously reported. These data also clearly establish that the cyclometalated 2-phenylpyridyl ligand is more electron-donating than the rollover bipy-H ligand.

The UV-Vis characterization of **1a** in CH_2_Cl_2_ shows two absorption bands at 358 and 328 nm (ε 3600 and 3400 L mol^−1^ cm^−1^, respectively) assigned to Pt→π* metal-to-ligand charge transfer (MLCT) processes on the complex mixed with intraligand transitions, and a typical ligand-based charge transfer at 268 nm (ε 20,500 L mol^−1^ cm^−1^). Analogously, in the case of **1b** in the CH_2_Cl_2_ solution, the UV-Vis spectrum shows MLCT processes mixed with π-π* transitions at 352 and 313 nm (ε 1500 and 7000 L mol^−1^ cm^−1^, respectively). Furthermore, a typical intense ligand-based π-π* transition is observed at 254 nm (ε 20,000 L mol^−1^ cm^−1^).

##### **2a** and **2b**

The CV response of **2a** and **2b** shows a reproducible, irreversible anodic process at Ep,a = 1.01 and 1.17 V, respectively, at a potential scan rate equal to 100 mV s^−1^, shifted to more anodic potential values at increasing scan rate, and a corresponding increase in the current peak value. Hence, also in this case, the bpy complex appears more difficult to oxidize, again in agreement with the higher stability of the HOMO and the lower electron-donating ability of the rollover-bpy ligand.

In the UV-Vis spectrum of **2a** in CH_2_Cl_2_, an MLCT is evident at 378 nm (ε 2000 L mol^−1^ cm^−1^), and a further absorption band at 286 nm (ε 12,000 L mol^−1^ cm^−1^) arising from π-π* transitions of the aromatic N^C ligand. The UV-Vis characterization of **2b** again shows a transition attributable to a metal-to-ligand charge transfer at 372 nm (ε 3000 L mol^−1^ cm^−1^). A maximum absorption at 260 nm (ε 16,000 L mol^−1^ cm^−1^) with a shoulder at 300 nm is present, attributable to a π-π* transition on the bipyridine ligand.

The comparison of voltammetric data (Table 5) of **1a-b** and **2a-b** demonstrates that, in all cases, the anodic process appears irreversible, presumably due to a (fast) chemical reaction following the charge transfer on the electrode. The irreversibility of anodic processes of Pt(II) derivatives is usually ascribed to the instability of the corresponding (formal) Pt(III) species [54]. The voltammetric behavior of the species investigated, in this study, suggests an electron-withdrawing effect of Cl ligand higher than for Me, making the metal center more electron-rich in the Me derivative than in the Cl derivative. This finding is in agreement with what we previously observed in [54] in comparable Pt(II) complexes where the cyclometalated ligand was 2-vinylpyridine. Moreover, the comparison with DFT calculations indicates that, in Cl derivatives, the highest occupied molecular orbital (HOMO) is located on the metal center, on the cyclometalating ligand and, to a minor extent, on the Cl ligand. Additionally, in the ppy-Me derivative, the HOMO is located on the Pt center and on the organic ligand, whereas in the bpy-Me complex, the HOMO is mainly located on the Pt center. Hence, the comparison of voltammetric and DFT results indicates that in the Cl derivatives reported here the oxidation process involves the metal center as well as the pyridine and the anionic ligands, while in **1a** and **1b**, the Me ligand is never involved in the process.

Cyclic voltammetry responses also allow an estimation of the energy value of HOMOs. In both couples of complexes (**1a-1b** and **2a-2b**) the higher anodic potential value of the Cl derivative corresponds to a more stabilized HOMO. In addition, the ppy derivatives are easier to oxidize than the corresponding bpy derivatives, showing a higher electron-donating ability of the ppy ligand as compared with the bpy cyclometalating ligand.

Eventually, the analysis of the energy gap (Eg) values derived both from the spectroscopic data and from the DFT calculations (Table 5) shows lower Eg values for Cl complexes (**2a** and **2b**). This feature suggests a higher charge density delocalization of **2a-b** as compared with **1a-b**, according to the higher stability of the Cl derivatives indicated by the voltammetric evidences. Furthermore, the composition of the frontiers molecular orbitals is also in agreement with this data. Indeed, in **2a-b**, the charge is extended over metal center, cyclometalating ligand, and chloride ligand, whereas in **1a-b**, only Pt and (mainly in **1a**) cyclometalating ligand are involved.

Small differences in the Eg values can be observed by comparing ppy and bpy derivatives. Although **1a** and **2a** show Eg values lower than the analogous **1b** and **2b**, respectively, the difference is quite small, hence, suggesting little influence of the cyclometalated ligand on the electronic delocalization.

## 3. Materials and Methods

All the solvents were purified and dried according to standard procedures [55]. The starting complex *cis*-[Pt(Me)_2_(DMSO)_2_] was synthesized according to [56,57,58]. Elemental analyses were performed with a Perkin-Elmer elemental analyzer 240B at the Department of Chemistry and Pharmacy of the University of Sassari or by the Warwick Analytical Service (University of Warwick, Coventry, UK).

The ^1^H, ^13^C, and ^31^P NMR spectra were recorded with Bruker Avance III 400, 500, or 600 spectrometers. Chemical shifts are given in ppm relative to internal TMS for ^1^H and ^13^C, and external 85% H_3_PO_4_ for ^31^P; J values are given in Hz. Two-dimensional spectra were obtained by means of standard pulse sequences.

Electrochemical characterizations were performed under argon atmosphere in a three-electrode, single compartment cell, with a CHI-650 potentiostat interfaced with a PC and using the specific software. A 2 mm diameter Pt disk was the working electrode, a Ag/AgCl with a suitable salt bridge was the reference electrode, and a graphite rod was the auxiliary electrode. The working electrode was polished with 1 and 0.3 μm alumina powder, and then rinsed with distilled water. All the experiments were carried out in CH_2_Cl_2_ (anhydrous, ≥99.8%, packaged under nitrogen), using 0.1 M tetraethylammonium hexafluorophosphate (TEAPF_6_) as supporting electrolyte, at a potential scan rate equal to 100 mV s^−1^. The concentration of complexes was, in each case, equal to 2 × 10^−3^ M. All potential values are finally referred to as the half-wave potential of the ferrocene/ferricinium (Fc/Fc^+^) ion redox couple, as measured in cyclic voltammetry tests in CH_2_Cl_2_ solution.

The UV-Vis spectra were recorded with a T80+ UV/Vis (PG Instrument Ltd., Lutterworth, U.K.) spectrophotometer using the software UV Win5 v 5.0.5. All the solutions were 2 × 10^−5^ M in CH_2_Cl_2_ as the solvent.

Energy-gap values (Eg) were evaluated from the λ_onset_ in the UV-Vis spectra. HOMO energy values were evaluated from the equation E_HOMO_ (eV) = −*e*(E_onset_ + 4.71) [59].

### 3.1. DFT Calculations

DFTand TD-DFT calculations were carried out using the PBE0 hybrid functional developed by Perdew, Burke, and Ernzerhof [60] and modified in its hybrid version by Adamo and Barone [61], with ZORA [62,63,64,65] using the ZORA-def2-SVP basis set for all atoms, except for Pt for which we used a segmented all-electron relativistically contracted (SARC) basis set [66] along with the RI-JONX approximation as implemented in the ORCA 4.2.1 package [67,68] Harmonic analysis at the same level of theory was carried out on the equilibrium geometries to confirm the nature of the minimum (i.e., the absence of imaginary frequencies) on the PES.

Interactions with the solvent were investigated using ORCA implementation of the solvation model based on density (SMD) developed by Truhlar et al. [69].

Preliminary potential energy surface scans were performed with the Firefly QC package [65,70], which is partially based on the GAMESS (US) [71] source code.

The localized molecular orbital-energy decomposition analysis (LMO-EDA) [72] was performed as implemented in GAMESS 2020 R2 [73].

The charge decomposition analysis (CDA) and extended charge decomposition analysis (ECDA) were performed using the generalized approach [74,75], as implemented in MultiWFN [76].

Images were generated using the following software: Gabedit [77], ORTEP 3 [78], POV-Ray tracer [79], and VMD [80].

### 3.2. X-ray

Single crystals of [Pt(ppy-H)(CH_3_)(PPh_3_)], **1a** (C_30_H_26_NPPt), were grown from a CH_2_Cl_2_ solution. A suitable crystal was selected and mounted on a glass fibre using Fomblin oil on an Oxford Diffraction Xcalibur Gemini diffractometer with a Ruby CCD detector. The crystal was kept at 150 K. The temperature of the crystal was controlled using an Oxford Cryosystems Cobra cooler.

Hydrogen atoms were added at calculated positions and refined using a riding model. Anisotropic displacement parameters were used for all non-H atoms; H-atoms were given an isotropic displacement parameter equal to 1.2 (or 1.5 for methyl H-atoms) times the equivalent isotropic displacement parameter of the atom to which they were attached. Using Olex2 [81], the structure was solved with the XS structure solution program using direct methods and refined with the ShelXL refinement package using least squares minimization [82]. Full details are provided in Supplementary Materials Table S2.

Single crystals of [Pt(ppy-H)(Cl)(PPh_3_)], **2a** (C_29_H_23_NPClPt) were grown from a CH_2_Cl_2_ solution. A suitable crystal was selected and mounted on a glass fiber using Fomblin oil on a Xcalibur Gemini diffractometer with a Ruby CCD area detector. The crystal was kept at 150 K during data collection. Using Olex2, the structure was solved with the XS structure solution program using direct methods and refined with the ShelXL refinement package using least squares minimization. Full details are provided in Supplementary Materials Table S3.

X-ray crystallographic data in CIF format have been deposited at the Cambridge Crystallographic Data Center as supplementary publication no. CCDC 963378 and 1546694.

## 4. Preparations

Complexes **1a** and **1b** were obtained by reaction of cis-[Pt(DMSO)_2_Me_2_] with the ligand (ppy or bpy) at reflux for 3 h in anhydrous toluene under nitrogen atmosphere. Subsequently, PPh_3_ was added to the hot solution in a 1:1 molar ratio and left to react for 30 min; then, the solution was concentrated to a small volume and treated with n-hexane to form a precipitate. The solid was filtered off, washed with n-hexane, and vacuum pumped to give the analytical sample as a yellow solid.

Complex **1a**. Yield 90%. ^1^H NMR (CDCl_3_): 0.79 (d with sat, 3H, ^3^J_P-H_ = 7.9 Hz, ^2^J_Pt-H_ = 83.5 Hz, CH_3_); 6.50 (ddd, 1H, J_H-H_ = 7.2, 5.7, 1.5 Hz); 7.19 (td, 1H, J_H-H_ = 7.8, 1.4 Hz); 7.34–7.44 (m, 10 H, aromatics); 7.66 (ddd, 1H, J_H-H_ = 7.6, 8.2, 1.7 Hz); 7.72 (dt, 1H, J_H-H_ = 7.9, 1.5 Hz); 7.75–7.85 (m, 8H, aromatics); 8.00 (ddd with sat, 1H, J_H-H_ = 5.4, 1.1 Hz; ^4^J_P-H_ = 7.1 Hz; J_Pt-H_ = 51 Hz, H adjacent to metalated C).

Selected ^13^C NMR (CDCl_3_): −11.41 (d with sat, ^1^J_Pt-C_ = 735 Hz, ^2^J_P-C_ = 5.0 Hz, Pt-*C*H_3_); 147.8 (s with sat, J_Pt-C_ = 10.5 Hz); 150.9 (d with sat, J_Pt-C_ = 16 Hz, J_P-C_ = 4.0 Hz); 164.2 (d with sat, ^1^J_Pt-C_ = 956 Hz, J_P-C_ = 120.5 Hz, Pt-C(sp^2^)); 167.1 (d with sat, J_Pt-C_ = 69 Hz, J_P-C_ = 5.8 Hz).

Complex **1b**. Yield 90%. Melting point: 215 °C. ^1^H NMR (600 MHz, acetone-d6, 298 K, ppm): 8.34 (d br, 1H, J_H-H_ = 4.3 Hz, H_6_); 8.32 (d br, 1H, J_H-H_ = 7.9 Hz, H3′); 8.12 (ddd sat, 1H, ^3^J_Pt-H_ = 48 Hz, J_H-H_ = 7.4, 5.5, 1.7 Hz, H4); 7.95 (td, 1H, J_H-H_ = 7.6, 1.5 Hz, H4′); 7.81-7.75 (m, 7H, H6′ + Ho(PPh_3_) or Hm(PPh_3_)); 7.53–7.47 (m, 9H, Hp(PPh_3_) + Hm(PPh_3_) or Ho(PPh_3_)); 7.21 (ddd sat, 1H, ^4^J_Pt-H_ = n.r. Hz, J_H-H_ = 7.4, 4.7, 1.7 Hz, H5); 6.85 (ddd, 1H, J_H-H_ = 7.4, 5.6, 1.4 Hz, H5′); 0.66 (d sat, 1H, ^2^J_Pt-H_ = 84.6 Hz, ^3^J_P-H_ = 7.7 Hz, Pt-CH_3_). ^13^C NMR (75.4 MHz, CDCl_3_, 298 K, ppm): 165.7 (s sat, ^2^J_Pt-C_ = 19.6 Hz, C2′); 164.7 (d sat, ^2^J_Pt-C_ = 48.3 Hz, _3_J_P-C_ = 3.5 Hz, C2); 155.4 (d sat, J_Pt-C_ = 970.3 Hz, ^2^J_P-C_ = 119.6 Hz, C3); 150.5 (d sat, ^3^J_Pt-C_ = 13.7 Hz, ^4^J_P-C_ = 3.8 Hz, C6′); 145.0 (s, C4′); 140.0 (s sat, ^2^J_Pt-C_ = 82.4 Hz, C4); 137.5 (s, C6); 135.0 (d sat, ^3^J_Pt-C_ = 17.1 Hz, ^2^J_P-C_ = 11.9 Hz, C*o*(PPh_3_)); 132.1 (d sat, ^2^J_Pt-C_ = 17.0 Hz, J_P-C_ = 44.0 Hz, H*ipso*(PPh_3_)); 130.3 (d, ^4^J_P-C_ = 1.7 Hz, H*p*(PPh_3_)); 126.3 (d, ^3^J_P-C_ = 9.8 Hz, H*m*(PPh_3_)); 124.5 (d sat, ^2^J_Pt-C_ = 53.4 Hz, ^3^J_P-C_ = 5.6 Hz, C5); 123.7 (s sat, ^3^J_Pt-C_ = 11.1 Hz, C5′); 121.4 (s sat, ^3^J_Pt-C_ = 20.1 Hz, C3′); −12.4 (d sat, J_Pt-C_ = 725.3 Hz, ^2^J_P-C_ = 4.7 Hz, Pt-CH_3_). ^31^P NMR (121.4 MHz, CDCl_3_, 298 K, ppm): 33.6 (s sat,^1^J_Pt-P_ = 2229 Hz, PPh_3_).

Complex **2a** was obtained following the procedure reported in [36].

Complex **2b** was obtained following three different procedures.

Method A. Reaction of [Pt(bpy-H)(Cl)(DMSO)] in CH_2_Cl_2_ with PPh_3_ in a 1:1 molar ratio (79.6 mg, 0.304 mmol, 1 eq). After 2 h, the mixture was concentrated to small volume and treated with n-hexane yielding the product as a yellow solid. Yield 90%. Taken from from [40].

Method B. To a solution of cis-[Pt(CH_3_)_2_(DMSO)_2_] (145.2 mg, 0.381 mmol, 1 eq) in anhydrous toluene (20 mL), bpy was added (122.3 mg, 0.783 mmol, 2.06 eq). The mixture was heated to reflux for 3 h and cooled to room temperature before the addition of [H_3_O 18-crown-6][BF_4_] (168.0 mg, 0.454 mmol, 1.2 eq) and 27.5 mg of LiCl (0.649 mmol, 1.7 eq) and 10 mL of acetone in order to solubilize everything. An orange precipitate formed and after 1 h 164.8 mg of PPh_3_ (0.628 mmol, 1.65 eq) were added. The precipitate dissolved and the solution became yellow, after 1 h, the mixture was filtered, concentrated to a small volume, and treated with Et_2_O and the yellow solid obtained filtered. Yield 65%.

Method C. To a solution of cis-[Pt(CH_3_)_2_(DMSO)_2_] (20.3 mg, 0.053 mmol, 1 eq) in anhydrous toluene (4 mL) were added 13.0 mg of bpy (0.083 mmol, 1.6 eq). The mixture was heated to reflux for 2 h, and then concentrated to approximately 2 mL before the addition of 530 µL of aqueous HCl 0.1 M (0.053 mmol, 1 eq) dissolved in 4 mL of acetone. Upon addition, the yellow solution became rapidly brownish and was left stirring overnight. The solution was extracted with CH_2_Cl_2_ (2 × 10 mL) and the chlorinated phase treated with Na_2_SO_4_, filtered, and concentrated. Addition of 16.4 mg of PPh_3_ (0.062 mmol, 1.2 eq) made the solution paler, evaporation of the solvent and treatment with Et_2_O gave the product. ^1^H and ^31^P NMR data were in agreement with those reported in [35]. 

## 5. Conclusions

Classical and rollover cyclometalated complexes are closely related, even though the latter family of derivatives possesses additional potentialities due to the uncoordinated donor atom. When two closely connected ligands are compared, such as 2-phenylpyridine and 2,2′-bipyridine, the corresponding Pt(II) cyclometalated complexes show a high level of general similarity. On the whole, when comparing Pt(II) electron-rich [Pt(N^C)Me(PPh_3_)] and electron-poor [Pt(N^C)Cl(PPh_3_)], the rollover-coordinated bpy-H ligand is demonstratably a worse donor than the cyclometalated ppy-H ligand, as shown, inter alia, by electrochemical data and DFT calculations. The rollover ligand, in addition, gives slightly more stable complexes. Noteworthy differences were found in the composition of frontier orbitals: The HOMO in the bpy-methyl complex **1b** essentially corresponds to the dz^2^ Pt orbital, whereas HOMO in **1a** is located on the Pt center and the C-bonded aromatic ring of the cyclometalated ppy-H ligand.

## Figures and Tables

**Table 1:** Distortion parameters for **1a-b** and **2a-b**. Improper torsion angles ω1 and ω2 and dihedral angles d.a. (deg), distances from planes in Å.

	1a′	1a″	1b	2a′	2a″	2a‴	2a′′′′	2b(mol1)	2b′(mol2)
CCDC				1546694	1457812	1505457	1505459		
ω1	−0.54 (9)	−1.5 (3)	+4.43 (8)	−1.34 (3)	−1.5 (1)	−3.8 (1)	+1.32 (6)	−1.09 (4)	−4.89 (4)
ω2	−3.12 (7)	−2.5 (2)	+4.01 (6)	+1.00 (4)	+0.7 (2)	−2.9 (1)	−1.04 (6)	−2.35 (5)	−2.57 (5)
Pl_1_/Pl_2_ d.a.	4.55	4.13	8.48	6.16	5.86	6.43	4.57	5.04	2.36
d.a. from Pl_3_									
Pl_4_(pyN)	9.91	9.90	10.82	0.73	0.71	0.83	6.91	4.02	7.84
Pl_5_(PyC)	7.91	7.41	10.29	5.44	5.25	5.62	4.43	5.98	7.72
Distance from Pl_1_ ^a^									
C1 or Cl	+0.028	+0.076	−0.227	+0.038	+0.056	−0.165	−0.058	−0.121	−0.143
P1	−0.180	−0.143	+0.233	+0.083	+0.073	+0.209	−0.072	+0.069	+0.264
d.a. between Pl_4_/Pl_5_ (PyN/PyC)	8.79	6.60	6.63	6.16	5.86	6.43	4.57	5.04	2.36

Planes Pl_1_–Pl_5_ as depicted in Figure 4. ^a^ Positive sign, position above the Pl_1_ plane as depicted in Figure 3. Negative sign, atom below the plane.

**Table 2:** ΔH and ΔG values between rotamers A and B of complexes **1a-b** and **2a-b** (kJ/mol, absolute values), vacuum and CH_2_Cl_2_ solution.

Complex	Gas-Phase		CH_2_Cl_2_	
	ΔH	ΔG	ΔH	ΔG
**1a**	0.31	1.33	2.13	1.72
**1b**	1.34	2.63	3.49	4.34
**2a**	8.60	7.62	8.58	9.44
**2b**	8.09	7.11	8.96	9.16

**Table 3:** Energy of the maximum for Pt-P and Pt-CH_3_ dihedral scan values in kJ/mol.

	Correct Isomer P-*trans*-C for 1a-b and P-*trans*-N for 2a-b	Inverted Isomer P-*trans*-N for 1a-b and P-*trans*-C for 2a-b
	Pt-P dihedral scan	
[Pt(bpy-H)(Cl)(PPh_3_)]	16.6	11.1
[Pt(ppy-H)(Cl)(PPh_3_)]	17.1	15.3
[Pt(bpy-H)(Me)(PPh_3_)]	11.7	14.8
[Pt(ppy-H)(Me)(PPh_3_)]	12.3	15.4
	Pt-CH_3_ dihedral scan	
Phenyl rings of PPh_3_	above/below bpy	above/below bpy
[Pt(bpy-H)(Me)(PPh_3_)]	5.0	5.1
[Pt(ppy-H)(Me)(PPh_3_)]	5.8	5.6

**Table 4:** ECDA and LMO-EDA results, DFT interaction energies are in kJ/mol.

	ECDA Results		DFT Interaction Energies				
	C^N ligand	PPh_3_ ligand	Me	Cl	bpy-H	ppy-H	PPh_3_
**1a**	−0.9335	−0.3237	−1075.55	-	-	−1236.82	−310.10
**1b**	−0.9304	−0.3330	−1093.57	-	−1213.46	-	−317.76
**2a**	−1.0335	−0.4565	-	−721.78	-	−1417.02	−422.58
**2b**	−1.0288	−0.4652	-	−735.67	−1390.62	-	−428.59

**Table 5:** Electrochemical, optical, and computational data.

Complex	WE = Pt CH_2_Cl_2_/TEAPF_6_ 0.1 M							
	E_ox_ (V)	HOMO (eV)	HOMO (eV) Theor	λ (nm) (ε, L mol^−1^ cm^−1^)	E_opt_ (eV)	E_theor_ (eV)	LUMO (eV)	LUMO (eV) Theor
**1a**	0.83	−5.65	−5.77 (gas) −6.16 (CH_2_Cl_2_)	268 (2.0 × 10^4^) 328 (3.4 × 10^3^) 358 (3.6 × 10^3^)	3.07	4.33	−2.54	−1.44ca (gas) −1.63 (CH_2_Cl_2_)
**2a**	1.01	−5.96	−5.68 (gas) −6.02 (CH_2_Cl_2_)	286 (1.2 × 10^4^) 378 (2.0 × 10^3^)	2.87	4.01	−2.86	−1.67 (gas) −1.76 (CH_2_Cl_2_)
**1b**	0.95	−5.67	−5.89 (gas) −6.28 (CH_2_Cl_2_)	254 (2.0 × 10^4^) 313 (7.0 × 10^3^) 352 (1.5 × 10^3^)	3.10	4.38	−2.28	−1.51 (gas) −1.72 (CH_2_Cl_2_)
**2b**	1.17	−6.00	−5.82 (gas) −6.14 (CH_2_Cl_2_)	258 (1.6 × 10^4^) 300^(S)^,372 (2.6 × 10^3^)	3.00	4.08	−2.46	−1.74 (gas) −1.86 (CH_2_Cl_2_)

E (V) vs. Fc/Fc+; WE = Pt; UV in CH_2_Cl_2_; LUMO = Eg,opt + HOMOex; E(HOMO) = −e(Eonset + 4.71) (eV).

## Data Availability

Data available from the corresponding author on request.

## Supplementary Materials

The following are available online at https://www.mdpi.com/article/10.3390/molecules27217249/s1, Figure S1: Expansion of the ^13^C NMR spectrum of **1a** and **2a**, Tables S1 and S2: Crystal data and structure refinement for **1a** and **2a**, Tables S3–S6: Coordinates of equilibrium geometries, Table S7: Orbital composition for selected MOs in the lowest energy conformer, Tables S8 and S9: CDA and ECDA results, Table S10: LMO-EDA results, Tables S11–S14: TD-DFT transitions.
