# Supplementary material for: Classical vs. Non-Classical Cyclometalated Pt(II) Complexes
Source: Molecules. 2022 Oct 25;27(21):7249. doi: 10.3390/molecules27217249 (PMC9654721; doi:10.3390/molecules27217249)
Supplement: Supplementary file 1 [file molecules-27-07249-s001.zip › molecules-1984325-supplementary.pdf]

Supplementary Materials

Expansion of the ^{13}C NMR spectrum of **1a** and **2a (**Figure S1)	page 2
Crystal data and structure refinement for **1a** (Table S1)	page 3
Crystal data and structure refinement for 2**a** (Table S2)	page 4
Coordinates of equilibrium geometries (Tables S3 to S6)	page 5
Orbital composition for selected MOs in the lowest energy conformer (Table S7)	page 13
CDA and ECDA results (Tables S8 and S9)	page 14
LMO-EDA results (Table S10)	page 15
TD-DFT transitions (Tables S11 to S14)	page 16

Figure S1. Expansion of the ^{13}C NMR spectrum of **1a** (above, red) and **2a** (below, blue) showing aromatic quaternary signals. A numbering scheme and a table are also given

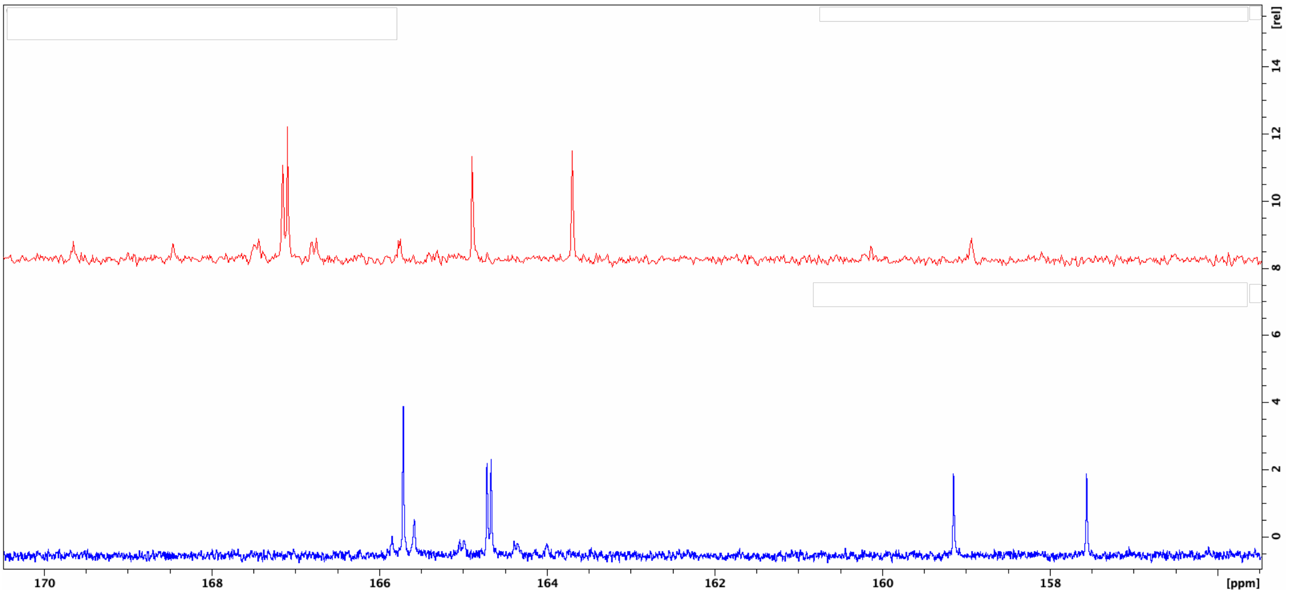

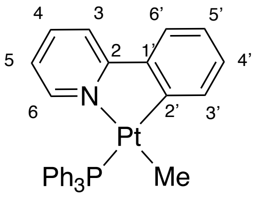

1a

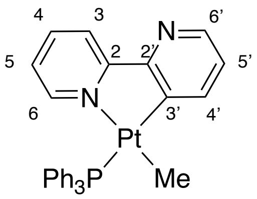

1b

Selected ^{13}C NMR signals for **1a** and **1b**.

	**1a**		**1b**
C2	167.1 (d sat, $^{2}J_{Pt\text{-}C}$ = 69 Hz, $^{3}J_{P\text{-}C}$ = 6.1 Hz)	C2	164.7 (d sat, $^{2}J_{Pt\text{-}C}$ = 48.5 Hz, $^{3}J_{P\text{-}C}$ = 3.5 Hz)
C6	123.8 or 135.5 d with sat	C6	150.5 (d sat, $^{3}J_{Pt\text{-}C}$ = 13.7 Hz, $^{4}J_{P\text{-}C}$ = 3.8 Hz)
C1′	147.8 (s sat, $^{2}J_{Pt\text{-}C}$ = 10.5 Hz)	C2′	165.7 (s sat, $^{2}J_{Pt\text{-}C}$ = 19.6 Hz)
C2′	164.2 (d sat, $^{1}J_{Pt\text{-}C}$ = 956 Hz, $J_{P\text{-}C}$ = 120.5 Hz)	C3′	158.3 (d sat, $J_{Pt\text{-}C}$ = 971 Hz, $^{2}J_{P\text{-}C}$ = 120 Hz)
C3′	132.5, s with sat $^{2}J_{Pt\text{-}C}$ = 85.5 Hz	C4′	140.0 (s sat, $^{2}J_{Pt\text{-}C}$ = 82.4 Hz)
C4′	129.9 d sat, $^{3}J_{P\text{-}C}$ = 7.2 Hz, $^{3}J_{Pt\text{-}C}$ = 60 Hz)	C5′	124.5 (d sat, $^{2}J_{Pt\text{-}C}$ = 53.4 Hz, $^{3}J_{P\text{-}C}$ = 5.6 Hz)

Table S1. Crystal data and structure refinement for **1a** and selected bond distances (in Å) and angles (in degrees) with corresponding ESDs in parentheses

Empirical formula	$C_{30}H_{26}NPPt$
Formula weight	626.58
Temperature (K)	150
Crystal system	Monoclinic
Space group	$P2_1/c$
a (Å)	9.52531(20)
b (Å)	13.0441(3)
c (Å)	19.4043(4)
α (°)	90
β (°)	100.161(2)
γ (°)	90
Volume ($Å^3$)	2372.16(9)
Z	4
ρcalc ($mg \cdot mm^{-3}$)	1.754
μ(Mo K_α) (mm^{-1})	5.999
F(000)	1224.0
Crystal size (mm^3)	0.2 x 0.08 x 0.06
2θ range for data collection	5.286 to 61.782°
Index ranges	$-13 \leq h \leq 13$, $-17 \leq k \leq 18$, $-26 \leq l \leq 27$
Reflections collected	61963
Independent reflections	7159 [R(int) = 0.0613]
Data/Restraints/Parameters	7159/0/299
Goodness-of-fit on F2	1.075
Final R indexes [$I \geq 2\sigma(I)$]	R_1 = 0.0322, wR_2 = 0.0658
Final R indexes [all data]	R_1 = 0.0430, wR_2 = 0.0695
Largest diff. peak/hole ($e \cdot Å^3$)	1.90/-1.16

Bond distances		Bond angles	
Pt-P	2.2955(9)	P-Pt-CH_3	91.8(1)
Pt-N	2.136(3)	C2′-Pt-CH_3	92.1(1)
Pt-C2′	2.044(3)	C2′-Pt-N	79.4(1)
Pt-CH_3	2.068(4)	N-Pt-P	96.69(8)
C2′-C1′	1.412(5)	P-Pt-C3	174.0(1)
C1′-C2	1.469(5)	N-Pt-CH_3	171.5(1)
C2-N1	1.359(5)		

Table S2. Crystal data and structure refinement for **2a** and selected bond distances (in Å) and angles (in degrees) with corresponding ESDs in parentheses

Empirical formula	$C_{29}H_{23}NPClPt$
Formula weight	646.99
Temperature (K)	150
Crystal system	Triclinic
Space group	P -1
a (Å)	9.89983(12)
b (Å)	10.22220(14)
c (Å)	13.5605(2)
α (°)	79.0363(12)
β (°)	70.6117(12)
γ (°)	67.7385(12)
Volume (Å^3)	1194.72(3)
Z	2
ϱcalc (mg·mm^{-3})	1.799
μ(Mo K_α) (mm^{-1})	6.069
F(000)	628.0
Crystal size (mm^3)	0.34 x 0.32 x 0.24
2θ range for data collection	5.556 to 30.58°
Index ranges	$-14 \leq h \leq 14$, $-15 \leq k \leq 14$, $-20 \leq l \leq 20$
Reflections collected	73315
Independent reflections	8197 [R(int) = 0.0315]
Data/Restraints/Parameters	6197/0/298
Goodness-of-fit on F2	1.096
Final R indexes [I ≥ 2σ(I)]	R_1 = 0.0141, wR_2 = 0.0319
Final R indexes [all data]	R_1 = 0.0151, wR_2 = 0.0321
Largest diff. peak/hole (e·Å^3)	0.64/-0.91

Bond distances		Bond angles	
Pt-P	2.2258(4)	P-Pt-Cl	91.55(2)
Pt-Cl	2.3757(6)	Cl-Pt-N1	92.60(4)
Pt-N1	2.086(1)	N1-Pt-C2′	80.46(6)
Pt-C2′	2.012(2)	C2′-Pt-P	95.34(5)
C2′-C1′	1.422(2)	P-Pt-N1	175.40(4)
C1′-C2	1.463(3)	Cl-Pt-C2′	172.93(5)
C2-N1	1.355(2)		

Table S3: Coordinates for the equilibrium geometry for [Pt(ppy-H)(Me)(PPh_3)], **1a**

Pt	-0.211786	-0.578951	0.282928
N	-1.436881	1.086173	0.885057
C	-2.734640	1.056523	1.211212
C	-3.468044	2.188882	1.536067
C	-2.816279	3.420539	1.517017
C	-1.473449	3.459958	1.174606
C	-0.787456	2.277826	0.857312
C	0.625559	2.219204	0.478150
C	1.133960	0.939643	0.137316
C	2.491672	0.877086	-0.211044
C	3.302767	2.010421	-0.232939
C	2.779819	3.262190	0.099903
C	1.441682	3.361907	0.456506
C	1.173379	-1.899158	-0.439284
P	-1.776495	-2.268217	0.585019
C	-3.354101	-1.944657	-0.317567
C	-4.623642	-2.312025	0.144949
C	-5.758215	-2.033620	-0.617554
C	-5.636864	-1.393139	-1.850708
C	-4.376264	-1.019988	-2.316940
C	-3.242085	-1.286100	-1.551444
C	-1.378877	-3.991489	0.072821
C	-0.410631	-4.694929	0.804477
C	-0.046988	-5.986241	0.433724
C	-0.638103	-6.589511	-0.678180
C	-1.594395	-5.893581	-1.414082
C	-1.964693	-4.599879	-1.041419
C	-2.269176	-2.535700	2.341266
C	-1.895205	-1.582773	3.297227
C	-2.249286	-1.748379	4.636873
C	-2.972992	-2.870979	5.035318
C	-3.330329	-3.837797	4.093571
C	-2.975540	-3.675559	2.755981
H	-3.217274	0.082405	1.205548
H	-4.520030	2.096075	1.793607
H	-3.348499	4.338328	1.762558
H	-0.946113	4.409164	1.144772
H	2.935175	-0.081230	-0.474503
H	4.352572	1.917944	-0.511944
H	3.410931	4.149580	0.084570
H	1.039875	4.338258	0.724367
H	1.982976	-2.031844	0.294767
H	1.616581	-1.480128	-1.352794
H	0.791458	-2.893457	-0.691899
H	-4.733377	-2.805205	1.108551
H	-6.741206	-2.319519	-0.245377
H	-6.524717	-1.178116	-2.443812
H	-4.274106	-0.510714	-3.273901
H	-2.256250	-0.968732	-1.895093
H	0.064034	-4.226447	1.666313

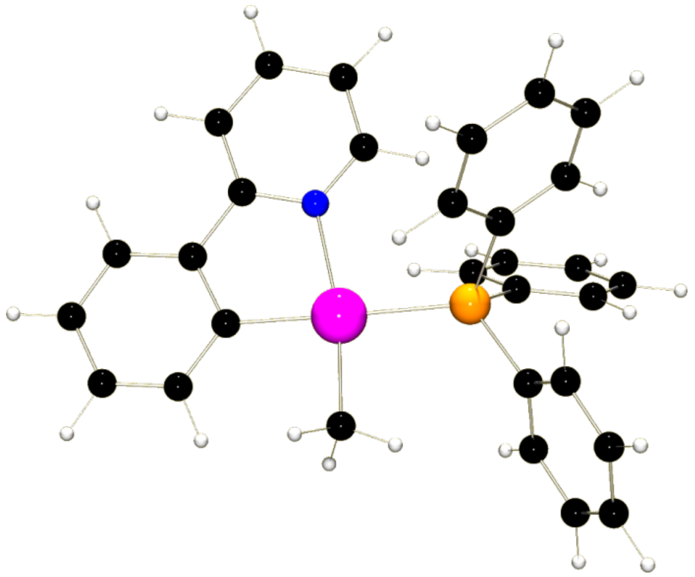

H	0.706228	-6.521088	1.010819
H	-0.350142	-7.598229	-0.969982
H	-2.058598	-6.356200	-2.283895
H	-2.715905	-4.066478	-1.621838
H	-1.302021	-0.723163	2.984406
H	-1.946974	-1.002355	5.370386
H	-3.247256	-3.001729	6.081430
H	-3.878297	-4.726775	4.403775
H	-3.228281	-4.450877	2.032800

Table S4: Coordinates for the equilibrium geometry for [Pt(ppy-H)(Cl)(PPh_3)], **1b**

Pt	-1.838309	0.238099	0.069735
N	-2.311481	2.262875	-0.122147
C	-3.450813	2.725326	-0.649183
C	-3.718462	4.083688	-0.747260
C	-2.763030	4.984187	-0.278126
C	-1.583370	4.497725	0.267411
C	-1.366437	3.115769	0.340336
C	-0.175869	2.466997	0.881690
C	-0.170797	1.043568	0.849394
C	0.983491	0.423296	1.356807
C	2.057314	1.150579	1.868506
C	2.023446	2.545439	1.896473
C	0.902663	3.197664	1.400012
Cl	-3.915365	-0.403502	-0.885528
P	-1.253128	-1.924195	0.338246
C	0.109554	-2.478221	-0.763331
C	0.890056	-1.528072	-1.432039
C	1.928105	-1.934666	-2.270446
C	2.192523	-3.291062	-2.451494
C	1.407477	-4.244915	-1.802344
C	0.366556	-3.842649	-0.968222
C	-0.724735	-2.298474	2.059397
C	-1.347517	-1.582754	3.090481
C	-1.034208	-1.851035	4.421770
C	-0.085829	-2.824765	4.734240
C	0.546484	-3.531284	3.711604
C	0.226496	-3.274120	2.378651
C	-2.528869	-3.210096	0.027094
C	-2.942164	-3.447016	-1.291645
C	-3.899567	-4.419303	-1.560894
C	-4.466068	-5.155725	-0.518688
C	-4.067841	-4.916417	0.793830
C	-3.099964	-3.947823	1.067545
H	-4.145759	1.957166	-0.990046
H	-4.657169	4.418291	-1.182022
H	-2.935995	6.058227	-0.337329
H	-0.825744	5.182418	0.638043
H	1.064646	-0.661646	1.367335
H	2.928861	0.619214	2.251386
H	2.859939	3.114670	2.298768
H	0.870963	4.286651	1.417960
H	0.670394	-0.469642	-1.297885
H	2.526379	-1.186051	-2.787467
H	3.002718	-3.607405	-3.107739
H	1.598107	-5.306602	-1.954659
H	-0.264718	-4.593639	-0.493906
H	-2.065285	-0.801487	2.837325
H	-1.523751	-1.288168	5.214769
H	0.166906	-3.027383	5.774253
H	1.295383	-4.285651	3.950591

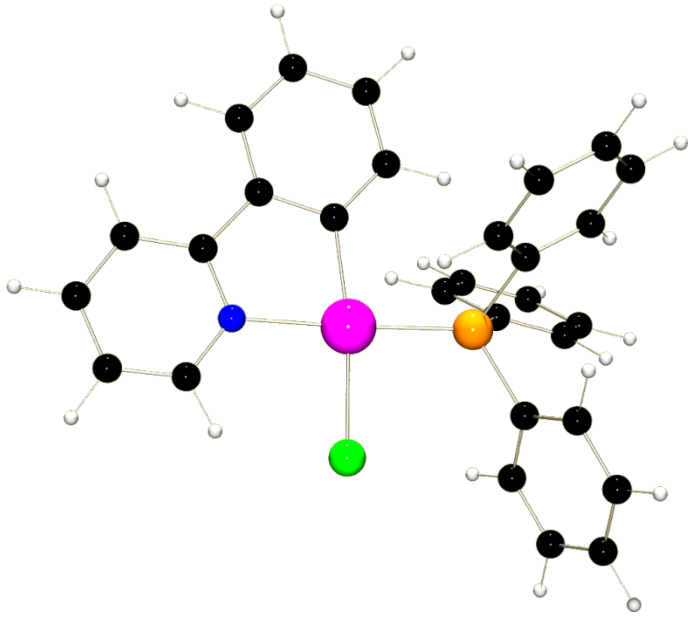

H	0.733927	-3.826349	1.589764
H	-2.521162	-2.862652	-2.107562
H	-4.214637	-4.594142	-2.588532
H	-5.219934	-5.912773	-0.732434
H	-4.507025	-5.484199	1.612625
H	-2.790845	-3.773084	2.096396

Table S5: Coordinates for the equilibrium geometry for [Pt(bpy-H)(Me)(PPh_3)], **2a**

Pt	-0.186857	-0.607068	0.323737
N	-1.424548	1.067148	0.920526
C	-2.720636	1.065929	1.256203
C	-3.416513	2.219045	1.596435
C	-2.736215	3.437159	1.587693
C	-1.394749	3.447363	1.235356
C	-0.763282	2.245589	0.902980
C	0.650620	2.181211	0.510883
C	1.159414	0.907325	0.172216
C	2.516304	0.899172	-0.182362
C	3.251063	2.082382	-0.192394
C	2.615028	3.276744	0.157690
N	1.337910	3.327074	0.505162
C	1.222642	-1.919635	-0.358685
P	-1.764978	-2.285058	0.601643
C	-3.333001	-1.931213	-0.304541
C	-4.606716	-2.304027	0.141930
C	-5.735324	-2.003830	-0.620926
C	-5.603858	-1.336056	-1.838525
C	-4.339035	-0.957811	-2.288704
C	-3.210573	-1.245989	-1.522675
C	-1.375915	-4.001220	0.061081
C	-0.439200	-4.738071	0.800793
C	-0.080423	-6.023073	0.403531
C	-0.644582	-6.586019	-0.742781
C	-1.569153	-5.856517	-1.486777
C	-1.934723	-4.569490	-1.087891
C	-2.268748	-2.570157	2.350843
C	-1.902553	-1.625432	3.317921
C	-2.268661	-1.801886	4.652893
C	-2.996752	-2.927196	5.035391
C	-3.347166	-3.885422	4.082370
C	-2.980797	-3.712248	2.749282
H	-3.229082	0.104751	1.246038
H	-4.468965	2.151941	1.861083
H	-3.247608	4.362280	1.850109
H	-0.792280	4.352368	1.200029
H	3.006819	-0.034647	-0.453995
H	4.304788	2.085710	-0.468739
H	3.164772	4.221018	0.156415
H	2.032666	-2.014126	0.381003
H	1.660723	-1.517942	-1.283058
H	0.861127	-2.928038	-0.583381
H	-4.724436	-2.817935	1.093710
H	-6.721690	-2.293830	-0.261157
H	-6.487192	-1.103867	-2.431836
H	-4.229001	-0.427383	-3.233241
H	-2.221426	-0.925189	-1.853350
H	0.013475	-4.302188	1.690953
H	0.647400	-6.584659	0.987836

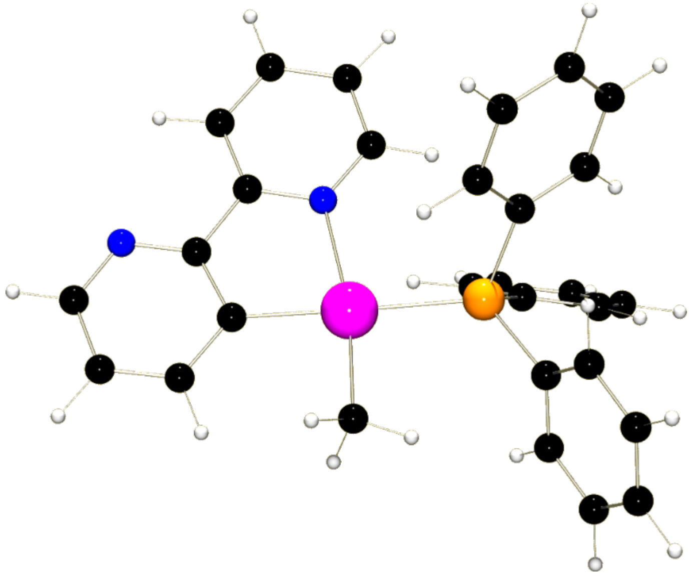

H	-0.360829	-7.589885	-1.054628
H	-2.012391	-6.287794	-2.383119
H	-2.661588	-4.009874	-1.674724
H	-1.306866	-0.762964	3.018178
H	-1.972618	-1.062073	5.395159
H	-3.280301	-3.066449	6.077886
H	-3.899298	-4.776023	4.380162
H	-3.229330	-4.480555	2.017249

Table S6: Coordinates for the equilibrium geometry for [Pt(bpy-H)(Cl)(PPh_3)], **2b**

Pt	-1.843521	0.219475	0.074218
N	-2.308659	2.255697	-0.104360
C	-3.435820	2.751172	-0.627622
C	-3.666113	4.119423	-0.700480
C	-2.693937	4.994762	-0.213725
C	-1.526510	4.473662	0.328058
C	-1.360301	3.089090	0.369492
C	-0.173603	2.423682	0.911319
C	-0.173934	1.004555	0.869010
C	0.994259	0.420273	1.386602
C	2.024112	1.211651	1.890344
C	1.882797	2.600558	1.875475
N	0.802158	3.194884	1.391755
Cl	-3.915408	-0.389094	-0.897710
P	-1.261727	-1.941433	0.335110
C	0.100896	-2.489195	-0.768318
C	0.885521	-1.535589	-1.427335
C	1.927069	-1.937773	-2.263547
C	2.190350	-3.293362	-2.452304
C	1.401030	-4.250457	-1.813116
C	0.357017	-3.852582	-0.980725
C	-0.722365	-2.311541	2.053367
C	-1.348853	-1.604819	3.088696
C	-1.023112	-1.868511	4.417917
C	-0.058816	-2.828605	4.724098
C	0.576597	-3.526442	3.697456
C	0.244470	-3.273924	2.366489
C	-2.537800	-3.225904	0.027371
C	-2.968000	-3.448021	-1.288539
C	-3.926434	-4.419916	-1.555955
C	-4.476611	-5.170001	-0.514775
C	-4.061288	-4.945288	0.795119
C	-3.092437	-3.977316	1.067143
H	-4.148266	2.007291	-0.984613
H	-4.595496	4.483272	-1.132523
H	-2.847834	6.072480	-0.257918
H	-0.723587	5.089578	0.726114
H	1.117294	-0.661399	1.408960
H	2.928551	0.756367	2.292465
H	2.671462	3.247048	2.265984
H	0.664283	-0.477784	-1.290442
H	2.528071	-1.186669	-2.773704
H	3.002824	-3.606440	-3.107226
H	1.590877	-5.311300	-1.971932
H	-0.276809	-4.605732	-0.513250
H	-2.080154	-0.834479	2.840786
H	-1.515840	-1.313054	5.214138
H	0.203514	-3.027462	5.762421
H	1.337454	-4.270227	3.931662
H	0.754187	-3.819287	1.574257

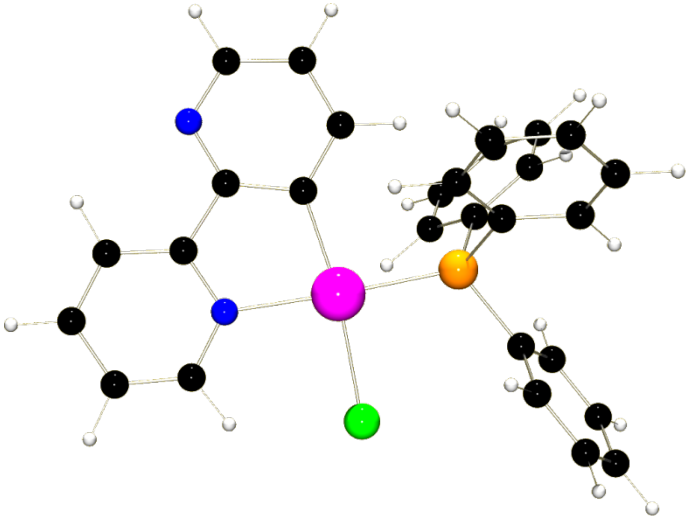

H	-2.560077	-2.852539	-2.103027
H	-4.255184	-4.583533	-2.581111
H	-5.231498	-5.926375	-0.727155
H	-4.487987	-5.523896	1.612920
H	-2.769749	-3.813722	2.093656

Supplementary Table S7: Orbital composition for selected MOs in the lowest energy conformer for each complex.

Complex	Orbital	Pt	bpy or ppy	PPh3	Cl or Me
[Pt(bpy-H)(Cl)(PPh3)]	HOMO-2	80.75%	9.78%	9.18%	0.30%
	HOMO-1	19.63%	7.68%	10.58%	62.09%
	HOMO	39.10%	32.56%	0.32%	27.99%
	LUMO	4.98%	91.54%	2.76%	0.72%
[Pt(ppy-H)(Cl)(PPh3)]	HOMO-2	85.34%	5.39%	7.60%	1.66%
	HOMO-1	19.96%	5.10%	12.31%	62.62%
	HOMO	38.86%	35.27%	0.34%	25.53%
	LUMO	5.89%	90.39%	2.93%	0.79%
[Pt(bpy-H)(Me)(PPh3)]	HOMO-2	75.78%	15.73%	0.62%	7.88%
	HOMO-1	41.83%	54.17%	3.19%	0.79%
	HOMO	80.22%	12.20%	1.68%	5.91%
	LUMO	6.18%	90.90%	2.61%	0.32%
[Pt(ppy-H)(Me)(PPh3)]	HOMO-2	72.71%	18.81%	0.50%	7.99%
	HOMO-1	49.15%	45.98%	3.14%	1.73%
	HOMO	72.22%	21.39%	1.77%	4.64%
	LUMO	7.03%	89.57%	3.01%	0.38%

Charge Decomposition and Extended Charge Decomposition Analysis

F1 and F2 indicate the first and second fragment, respectively.
d is the number of electrons donated from fragment 1 to fragment 2.
b is the number of electrons back donated from fragment 2 to fragment 1.
r is the number of electrons involved in repulsive polarization.

Table S8: Full CDA and ECDA results obtained using F1 = [Pt(X)(PPh_3)], X = Me, Cl, and F2 = cyclometalated ligand

	**1a**	**2a**	**1b**	**2b**
d	0.076195	0.076781	0.126594	0.127578
b	0.449513	0.453434	0.453612	0.461711
d-b	-0.373318	-0.376653	-0.327018	-0.334132
r	-0.497547	-0.486795	-0.485864	-0.473001
PL(1) + CT(1→2)	0.3531	0.3482	0.4742	0.4698
PL(2) + CT(1→2)	0.2372	0.2304	0.3238	0.3177
PL(1) + CT(2→1)	1.2866	1.2786	1.5077	1.4986
PL(2) + CT(2→1)	1.1706	1.1608	1.3573	1.3465
net e- obtained by F2	-0.9335	-0.9304	-1.0335	-1.0288

Table S9: Full CDA and ECDA results obtained using F1 = [Pt(NC)(PPh_3)], NC = ppy-H, bpy-H, and F2 = PPh_3

	**1a**	**2a**	**1b**	**2b**
d	0.130215	0.127743	0.146065	0.144899
b	0.311970	0.312776	0.309990	0.302886
d-b	-0.181755	-0.185033	-0.154934	-0.157988
r	-0.433162	-0.428164	-0.423603	-0.413929
PL(1) + CT(1→2)	0.2856	0.2775	0.2971	0.2979
PL(2) + CT(1→2)	0.2349	0.2319	0.2691	0.2679
PL(1) + CT(2→1)	0.6092	0.6106	0.7535	0.7630
PL(2) + CT(2→1)	0.5585	0.5649	0.7255	0.7330
net e- obtained by F2	-0.3237	-0.3330	-0.4565	-0.4652

Localized Molecular Orbital-Energy Decomposition Analysis (LMO-EDA)

ES is the electrostatic energy.
EX is the exchange energy.
REP is the repulsion energy.
POL is the polarisation energy.
DISP is the dispersion energy.
E is the total interaction energy (HF or DFT).

Table S10: Full LMO-EDA results in kJ/mol. In the column "Fragment" we indicated only one of the two parts of the partitioned molecule as all other atoms in the molecule were assigned to the second one.

		**ES=**	**EX=**	**REP=**	**POL=**	**DISP=**	**E=**
	Me	-658.69	-403.26	1209.60	-1125.51	-97.69	-1075.55
**1a**	ppy	-759.18	-459.04	1355.31	-1236.31	-137.60	-1236.82
	PPh3	-198.54	-310.90	908.19	-603.58	-105.28	-310.11
	Cl	-503.79	-177.18	490.48	-470.78	-60.51	-721.79
**1b**	ppy	-843.40	-506.98	1505.61	-1415.39	-156.86	-1417.02
	PPh3	-184.88	-327.69	969.40	-766.04	-113.37	-422.58
	Me	-668.72	-404.80	1208.70	-1131.48	-97.26	-1093.57
**2a**	bpy	-749.79	-453.02	1337.80	-1213.48	-134.96	-1213.46
	PPh3	-194.86	-306.68	894.49	-606.40	-104.31	-317.76
	Cl	-511.38	-176.70	488.55	-475.95	-60.19	-735.67
**2b**	bpy	-832.68	-499.69	1483.66	-1387.57	-154.35	-1390.63
	PPh3	-187.01	-328.74	974.45	-774.45	-112.82	-428.58

Table S11: Absorption spectrum via transition electric dipole moments and TD-DFT transitions (main three) for the 20 lowest excited states (singlets) [Pt(ppy-H)(Me)(PPh_3)], **1a**

State	Energy (cm-1)	Wavelength (nm)	fosc	Weight of individual excitations (printed three largest only)
1	28069.1	356.3	0.005647	153 -> 154 (88.0%), 152 -> 154 (7.4%), 151 -> 154 (1.7%)
2	29015.8	344.6	0.013496	152 -> 154 (85.9%), 151 -> 154 (7.6%), 153 -> 154 (4.7%)
3	31239.7	320.1	0.094791	151 -> 154 (85.1%), 152 -> 154 (5.1%), 153 -> 154 (3.8%)
4	33767.8	296.1	0.019196	153 -> 155 (31.4%), 150 -> 154 (27.8%), 153 -> 156 (25.9%)
5	34589.9	289.1	0.006394	149 -> 154 (54.5%), 150 -> 154 (22.4%), 152 -> 155 (9.2%)
6	35008.0	285.6	0.000470	148 -> 154 (83.5%), 152 -> 156 (3.5%), 153 -> 156 (3.0%)
7	35213.3	284.0	0.002387	152 -> 155 (59.3%), 149 -> 154 (15.9%), 152 -> 156 (8.6%)
8	35345.2	282.9	0.003120	152 -> 156 (34.0%), 153 -> 155 (26.9%), 153 -> 156 (26.0%)
9	36028.3	277.6	0.003908	152 -> 156 (45.9%), 152 -> 155 (23.6%), 153 -> 156 (14.7%)
10	36269.2	275.7	0.115815	150 -> 154 (18.9%), 151 -> 155 (17.6%), 151 -> 156 (15.9%)
11	36924.4	270.8	0.099579	151 -> 155 (62.5%), 150 -> 154 (15.7%), 153 -> 156 (5.3%)
12	37445.3	267.1	0.017479	151 -> 156 (30.8%), 153 -> 157 (27.9%), 152 -> 157 (24.5%)
13	37554.8	266.3	0.036663	151 -> 156 (40.8%), 153 -> 157 (33.3%), 152 -> 157 (7.7%)
14	38285.4	261.2	0.021208	152 -> 157 (54.7%), 153 -> 157 (27.6%), 152 -> 158 (3.8%)
15	38407.2	260.4	0.011424	153 -> 158 (49.3%), 152 -> 158 (26.4%), 152 -> 157 (5.0%)
16	39127.4	255.6	0.008543	152 -> 158 (51.2%), 153 -> 158 (25.8%), 152 -> 159 (3.8%)
17	39503.0	253.1	0.004705	151 -> 157 (64.8%), 143 -> 154 (11.7%), 153 -> 157 (2.8%)
18	39761.1	251.5	0.001187	143 -> 154 (49.0%), 151 -> 157 (15.8%), 140 -> 154 (7.7%)
19	39765.9	251.5	0.017976	149 -> 155 (57.5%), 150 -> 155 (18.6%), 149 -> 156 (7.0%)
20	40000.7	250.0	0.012711	153 -> 159 (39.7%), 152 -> 159 (11.6%), 153 -> 158 (11.0%)

Table S12: Absorption spectrum via transition electric dipole moments and TD-DFT transitions (main three) for the 20 lowest excited states (singlets) [Pt(ppy-H)(Cl)(PPh_3)], **1b**

State	Energy (cm-1)	Wavelength (nm)	fosc	Weight of individual excitations (printed three largest only)
1	26802.3	373.1	0.047824	157 -> 158 (97.3%)
2	31474.2	317.7	0.007071	154 -> 158 (7.0%), 155 -> 158 (55.9%), 156 -> 158 (31.1%)
3	31965.8	312.8	0.000680	156 -> 158 (59.6%), 155 -> 158 (27.8%), 157 -> 161 (4.0%)
4	32475.3	307.9	0.003133	157 -> 159 (18.9%), 157 -> 161 (54.7%), 157 -> 167 (10.2%)
5	33020.9	302.8	0.012030	157 -> 159 (46.8%), 157 -> 160 (16.4%), 157 -> 161 (17.0%)
6	34346.9	291.1	0.025924	154 -> 158 (18.3%), 157 -> 159 (27.1%), 157 -> 160 (43.6%)
7	34544.7	289.5	0.065278	151 -> 158 (5.9%), 154 -> 158 (43.5%), 157 -> 160 (36.2%)
8	36441.3	274.4	0.024023	155 -> 161 (45.2%), 155 -> 167 (12.9%), 156 -> 161 (10.8%)
9	36843.4	271.4	0.061254	157 -> 162 (57.0%), 151 -> 158 (21.8%), 154 -> 159 (2.7%)
10	37015.4	270.2	0.071479	151 -> 158 (36.3%), 157 -> 162 (32.2%), 156 -> 161 (10.5%)
11	37243.5	268.5	0.036406	156 -> 161 (42.8%), 155 -> 161 (13.4%), 156 -> 159 (9.9%)
12	38091.3	262.5	0.017244	152 -> 158 (34.1%), 153 -> 158 (25.0%), 150 -> 158 (11.3%)
13	38320.7	261.0	0.003817	156 -> 159 (52.8%), 155 -> 159 (18.9%), 156 -> 161 (7.1%)
14	38533.2	259.5	0.014251	157 -> 163 (71.2%), 153 -> 158 (15.3%), 151 -> 158 (2.3%)
15	38691.0	258.5	0.002190	155 -> 159 (46.9%), 156 -> 159 (21.5%), 155 -> 160 (12.0%)
16	38731.3	258.2	0.004123	153 -> 158 (34.5%), 152 -> 158 (23.7%), 156 -> 160 (8.8%)
17	38990.7	256.5	0.004286	156 -> 160 (54.5%), 155 -> 160 (17.1%), 153 -> 158 (7.1%)
18	39524.6	253.0	0.000979	145 -> 158 (26.7%), 146 -> 158 (25.5%), 148 -> 158 (20.7%)
19	39635.6	252.3	0.009314	155 -> 160 (52.9%), 156 -> 160 (21.1%), 155 -> 159 (6.3%)
20	39795.8	251.3	0.029801	157 -> 164 (75.2%), 154 -> 159 (4.3%), 155 -> 160 (2.8%)

Table S13: Absorption spectrum via transition electric dipole moments and TD-DFT transitions (main three) for the 20 lowest excited states (singlets) [Pt(bpy-H)(Me)(PPh_3)], **2a**

State	Energy (cm-1)	Wavelength (nm)	fosc	Weight of individual excitations (printed three largest only)
1	28271.5	353.7	0.004100	153 -> 154 (78.7%), 152 -> 154 (18.1%)
2	28868.2	346.4	0.009715	152 -> 154 (74.3%), 153 -> 154 (13.9%), 151 -> 154 (8.6%)
3	31369.4	318.8	0.093542	151 -> 154 (85.5%), 152 -> 154 (4.8%), 153 -> 154 (3.3%)
4	31770.3	314.8	0.004808	150 -> 154 (76.3%), 149 -> 154 (11.9%), 147 -> 154 (2.6%)
5	34362.3	291.0	0.020754	153 -> 155 (52.9%), 152 -> 155 (29.8%), 153 -> 156 (3.4%)
6	34799.4	287.4	0.018629	149 -> 154 (35.4%), 152 -> 155 (24.7%), 153 -> 155 (19.1%)
7	35057.7	285.2	0.013252	149 -> 154 (42.3%), 152 -> 155 (27.6%), 150 -> 154 (10.4%)
8	35845.1	279.0	0.001445	153 -> 156 (57.7%), 152 -> 156 (29.9%), 153 -> 155 (4.2%)
9	36467.9	274.2	0.018412	151 -> 155 (84.3%), 153 -> 155 (3.3%), 151 -> 156 (1.9%)
10	36610.0	273.1	0.007716	152 -> 156 (55.1%), 153 -> 156 (26.4%), 151 -> 156 (5.8%)
11	37029.7	270.1	0.000708	150 -> 155 (51.3%), 149 -> 155 (5.7%), 150 -> 156 (4.6%)
12	37861.1	264.1	0.021522	153 -> 157 (38.1%), 152 -> 157 (27.4%), 151 -> 156 (13.5%)
13	37948.3	263.5	0.027658	151 -> 156 (59.9%), 148 -> 154 (14.1%), 153 -> 157 (7.4%)
14	38421.0	260.3	0.142547	148 -> 154 (51.2%), 151 -> 156 (13.8%), 151 -> 157 (3.8%)
15	38691.9	258.5	0.016454	152 -> 158 (27.5%), 153 -> 158 (27%), 153 -> 157 (17.4%)
16	38762.7	258.0	0.045421	152 -> 157 (50.4%), 153 -> 157 (17.2%), 153 -> 158 (11.6%)
17	39151.9	255.4	0.001524	147 -> 154 (20.0%), 145 -> 154 (15.6%), 141 -> 154 (15.4%)
18	39519.2	253.0	0.008125	152 -> 158 (33.2%), 153 -> 158 (17.8%), 150 -> 156 (13.5%)
19	39709.2	251.8	0.009537	150 -> 156 (30.3%), 142 -> 154 (17.2%), 141 -> 154 (7.5%)
20	39742.0	251.6	0.007977	150 -> 156 (26.4%), 142 -> 154 (22.2%), 141 -> 154 (10.1%)

Table S14: Absorption spectrum via transition electric dipole moments and TD-DFT transitions (main three) for the 20 lowest excited states (singlets) [Pt(bpy-H)(Cl)(PPh_3)], **2b**

State	Energy (cm-1)	Wavelength (nm)	fosc	Weight of individual excitations (printed three largest only)
1	27113.2	368.8	0.052919	157 -> 158 (96.7%)
2	30928.5	323.3	0.001177	155 -> 158 (46.7%), 156 -> 158 (44.3%), 153 -> 158 (3.8%)
3	31454.0	317.9	0.004467	155 -> 158 (47.4%), 156 -> 158 (42.4%), 153 -> 158 (6.6%)
4	32730.9	305.5	0.004743	157 -> 161 (59.1%), 157 -> 159 (20.5%), 157 -> 167 (11.2%)
5	33418.7	299.2	0.021351	157 -> 159 (71.4%), 157 -> 161 (17.3%), 157 -> 160 (3.6%)
6	33968.4	294.4	0.002208	153 -> 158 (49.0%), 152 -> 158 (22.6%), 151 -> 158 (7.2%)
7	35147.0	284.5	0.000204	157 -> 160 (92.3%), 157 -> 159 (2.3%), 157 -> 161 (2.0%)
8	35488.4	281.8	0.020961	154 -> 158 (58.0%), 155 -> 161 (8.8%), 150 -> 158 (6.2%)
9	36513.8	273.9	0.037419	155 -> 161 (50.7%), 155 -> 167 (12.5%), 154 -> 158 (7.5%)
10	36916.2	270.9	0.005991	156 -> 161 (53.2%), 156 -> 159 (18.5%), 156 -> 167 (7.0%)
11	37253.1	268.4	0.183019	150 -> 158 (29.6%), 154 -> 158 (19.6%), 151 -> 158 (17.7%)
12	37568.4	266.2	0.005105	156 -> 159 (36.4%), 155 -> 159 (14.8%), 156 -> 161 (14.0%)
13	37621.5	265.8	0.029671	157 -> 162 (77.0%), 150 -> 158 (2.8%), 156 -> 159 (2.2%)
14	38026.0	263.0	0.005474	155 -> 159 (63.1%), 156 -> 159 (17.1%), 155 -> 161 (4.2%)
15	38238.8	261.5	0.001392	152 -> 158 (60.2%), 153 -> 158 (20.1%), 156 -> 159 (4.6%)
16	38850.3	257.4	0.049928	157 -> 163 (69.5%), 157 -> 164 (7.6%), 157 -> 162 (4.9%)
17	39263.6	254.7	0.003967	156 -> 160 (60.3%), 155 -> 160 (32.5%), 156 -> 161 (1.2%)
18	39604.1	252.5	0.005049	155 -> 160 (51.7%), 156 -> 160 (20.8%), 157 -> 163 (4.5%)
19	39654.9	252.2	0.002459	149 -> 158 (27.5%), 151 -> 158 (21.8%), 157 -> 163 (8.2%)
20	40017.6	249.9	0.001178	153 -> 159 (30.7%), 152 -> 159 (12.9%), 156 -> 160 (8.2%)
